# Supplementary material for: Voluntary wheel running exercise rescues behaviorally-evoked acetylcholine efflux in the medial prefrontal cortex and epigenetic changes in ChAT genes following adolescent intermittent ethanol exposure
Source: PLoS One. 2024 Oct 22;19(10):e0311405. doi: 10.1371/journal.pone.0311405 (PMC11495633; doi:10.1371/journal.pone.0311405)
Supplement: S1 File — Raw data for all analyses, with subjects identified by ID number, sex, litter, gavage treatment, and exercise condition. (PDF) [file pone.0311405.s003.pdf]

Animal ID	Treatment	Sex	Gavage1	Gavage2	Gavage3	Gavage4	Gavage5	Gavage6	Gavage7
101	AIE	F	80	81	101	105	121	125	148
104	Con	M	86	93	113	117	140	147	168
105	Con	M	70	77	94	102	121	129	145
107	Con	F	80	86	104	108	127	129	146
108	Con	F	76	81	95	99	113	121	135
109	AIE	M	86	88	109	112	136	140	165
113	AIE	F	88	90	109	118	134	137	147
114	Con	M	100	109	133	143	168	178	207
116	Con	M	102	112	135	147	171	178	208
118	AIE	M	97	100	124	127	154	159	187
121	Con	F	92	100	119	125	142	151	160
123	Con	F	99	105	130	135	154	161	171
124	AIE	M	90	94	116	124	150	160	183
127	Con	M	94	102	126	134	160	167	193
128	Con	F	84	93	114	124	140	143	154
129	Con	F	84	92	111	118	135	136	153
131	AIE	M	97	102	128	135	159	169	192
132	AIE	M	95	100	124	133	162	168	196
134	AIE	F	84	86	105	112	125	130	143
137	Con	F	76	80	100	106	128	135	151
138	Con	F	80	89	108	115	142	148	163
139	AIE	F	79	80	101	106	127	135	152
140	AIE	F	80	81	102	108	127	134	156
141	Con	M	87	93	119	127	155	167	195
144	Con	F	76	84	102	109	131	139	159
146	AIE	M	85	89	115	124	147	158	188
148	Con	M	89	96	118	127	154	160	187
149	Con	M	92	98	123	128	156	162	189
151	AIE	F	70	72	88	95	109	117	129
153	AIE	F	85	88	107	116	135	142	161
154	AIE	M	87	90	113	120	152	158	185
156	AIE	M	92	94	109	115	143	151	178
157	Con	F	80	87	108	117	136	143	164
158	Con	F	82	86	106	115	135	142	155
160	AIE	M	95	100	123	132	154	162	187
165	Con	M	90	98	120	128	153	123	186
167	AIE	F	86	93	108	114	167	136	154
168	AIE	F	87	92	113	117	143	146	164
172	AIE	M	77	82	105	110	136	144	165
173	AIE	M	71	77	99	102	127	130	155
174	AIE	F	73	78	92	97	117	123	138
175	AIE	F	67	70	90	93	111	116	134
177	Con	M	80	86	108	115	139	148	175
178	Con	M	74	80	105	110	136	143	172
180	Con	F	69	76	93	95	119	123	137
185	AIE	M	82	89	112	122	148	152	174

186	AIE	F	73	78	100	105	127	132	148
187	AIE	F	78	83	101	108	132	137	157
191	Con	M	86	94	118	127	157	171	198
192	Con	F	72	79	100	104	127	135	148
197	AIE	M	62	66	84	88	109	113	133
198	AIE	F	67	76	91	92	112	114	128
199	AIE	F	57	66	73	75	93	94	109
201	Con	M	73	76	94	100	123	128	150
205	Con	F	59	61	80	80	103	105	120
206	Con	F	63	66	85	86	106	109	120
212	AIE	M	63	64	81	85	104	110	133
213	Con	F	61	65	81	87	107	108	123
214	Con	F	59	61	79	82	102	109	123
216	Con	M	61	63	81	80	108	116	135
217	Con	M	60	63	82	85	106	112	132
220	AIE	M	72	81	93	105	117	130	146
223	Con	M	83	87	110	119	145	150	170
226	Con	F	74	77	87	96	109	113	126
227	Con	F	74	73	91	94	112	117	131
229	AIE	M	72	73	91	94	115	121	140
230	AIE	F	69	71	87	91	109	111	130
233	Con	M	73	78	99	102	123	129	153
301	Con	F	58	64	79	84	119	124	107
302	Con	F	54	61	75	78	108	113	124
303	AIE	F	60	62	76	81	112	119	125
305	Con	M	62	65	84	90	130	137	155
306	Con	M	67	72	91	98	142	148	166
308	AIE	M	60	67	82	87	122	129	147
309	AIE	M	63	69	84	90	132	139	158
311	Con	M	66	73	88	95	112	123	137
312	Con	F	66	71	89	94	108	119	135
313	Con	F	68	72	88	90	111	114	127
314	Con	M	72	77	98	101	123	129	150
315	AIE	M	72	78	96	97	120	125	148
316	AIE	M	75	76	101	106	135	140	167
318	Con	F	65	72	85	92	106	113	127
322	AIE	F	69	70	89	89	108	113	132
339	Con	M	73	80	100	106	128	137	159
340	Con	M	71	75	98	100	120	129	151
345	Con	M	77	85	103	109	134	141	159
347	AIE	M	74	79	100	102	132	136	157
348	AIE	M	79	86	110	117	142	147	169
349	Con	F	73	80	96	101	121	126	139
350	Con	F	65	70	82	91	111	116	132
351	AIE	F	64	69	80	86	101	106	120
352	AIE	F	81	88	108	115	136	140	157
361	Con	M	82	90	109	116	140	147	168

362	AIE	M	84	89	111	113	140	143	165
363	AIE	F	73	74	91	98	116	119	130
364	AIE	F	66	72	85	91	110	113	133
365	Con	F	69	72	87	95	117	120	135
366	Con	F	73	77	96	100	126	124	140
379	AIE	F	73	77	93	92	106	116	132
380	AIE	F	67	70	87	88	107	112	127
383	AIE	M	89	96	120	127	150	160	182
384	AIE	M	66	70	91	95	117	121	142
387	AIE	F	67	68	90	89	104	114	130
388	AIE	F	61	63	79	79	94	102	119
389	Con	F	68	72	93	101	117	128	143
390	Con	F	77	85	105	106	127	132	148
391	Con	F	67	75	92	98	117	123	138
392	Con	F	69	74	94	95	116	120	136

Gavage8	Gavage9	Gavage10	Gavage11	Gavage12	Gavage13	Gavage14	Gavage15	Gavage16
146	160	164	180	183	198	194	210	210
172	157	194	213	216	236	245	272	274
151	156	179	196	205	224	236	261	265
148	184	154	171	178	189	193	198	203
138	185	151	165	170	181	186	194	196
167	188	185	206	205	230	229	251	253
152	167	170	176	180	194	191	201	205
213	240	251	274	284	318	321	345	350
213	243	252	276	288	318	322	346	348
192	220	225	251	256	278	279	299	299
168	178	189	191	195	203	208	207	207
179	191	197	203	210	220	229	233	232
188	220	223	252	258	279	284	303	308
203	232	241	272	279	311	314	338	344
160	176	183	191	195	209	210	215	215
153	171	174	188	191	201	203	209	210
199	230	235	263	269	297	302	317	322
203	238	242	266	269	303	302	315	324
146	154	160	169	174	183	186	195	195
155	170	170	185	188	200	200	207	218
170	181	183	198	198	215	216	224	231
154	175	173	189	195	206	206	214	221
156	175	178	187	192	206	211	204	217
207	236	247	275	281	315	328	358	361
167	181	189	204	201	221	220	231	235
198	228	238	264	274	305	313	341	348
194	224	231	257	266	289	291	323	328
200	220	237	268	272	299	313	342	346
135	151	153	164	164	175	172	219	179
167	183	188	198	205	220	219	257	227
190	222	224	255	261	293	292	310	317
181	208	212	240	241	275	276	292	310
166	179	182	196	198	214	222	233	237
165	182	191	198	202	220	228	234	239
198	222	224	255	260	292	256	318	319
197	218	224	254	259	284	282	314	326
162	173	180	194	195	209	206	215	223
169	179	185	198	200	218	222	233	240
175	197	206	229	232	261	267	288	296
160	182	185	214	215	240	247	265	272
144	157	160	172	175	186	185	196	202
139	154	156	170	172	183	182	199	202
183	203	213	244	249	279	283	311	321
182	203	211	240	244	270	277	306	313
148	159	163	172	177	185	188	197	199
186	205	208	236	237	268	267	297	304

157	170	176	182	190	197	200	206	212
162	180	180	198	201	213	216	225	228
207	239	245	276	283	312	312	340	351
159	174	173	183	181	197	188	208	208
138	155	158	170	170	194	199	214	222
132	146	145	164	164	169	177	187	190
115	130	129	138	145	154	160	164	172
158	172	175	199	203	219	233	251	258
125	138	132	143	152	160	165	177	178
123	129	128	142	145	156	163	170	176
138	159	163	186	170	207	209	222	234
130	143	147	156	167	171	179	185	190
129	148	148	164	170	180	192	196	205
142	162	168	188	202	215	230	245	255
136	158	162	186	200	212	230	241	252
150	169	177	197	209	232	235	264	260
176	209	216	237	247	274	278	301	309
127	142	147	157	159	172	171	175	175
134	149	150	155	158	168	167	169	173
138	168	176	199	201	218	221	245	241
129	145	145	163	175	183	185	206	206
159	175	189	210	223	236	244	270	270
142	151	153	169	168	175	185	184	188
128	142	142	153	154	158	163	172	170
128	143	140	156	151	165	160	173	170
161	179	186	203	209	221	240	258	255
172	191	200	222	226	233	247	267	266
154	170	177	191	196	218	220	245	286
162	183	186	209	210	232	240	261	264
145	158	162	184	188	207	214	230	238
139	146	149	162	167	175	179	188	189
133	141	141	154	158	163	168	174	180
156	176	183	202	207	228	232	252	253
155	171	177	196	207	230	230	255	259
168	185	195	221	228	251	251	278	279
133	144	148	157	165	172	178	182	190
133	150	149	166	169	182	182	193	198
170	191	197	223	225	251	253	275	283
160	177	182	209	210	238	241	259	277
165	186	192	216	219	239	241	268	277
158	182	181	206	209	230	230	257	261
171	193	195	225	227	251	252	279	280
141	157	157	169	172	180	184	197	188
136	148	149	163	161	173	175	179	187
127	141	145	155	159	172	174	184	183
159	176	170	193	191	206	207	222	218
173	192	196	215	222	238	247	264	269

169	191	190	217	221	240	244	273	277
138	151	153	168	172	182	183	187	194
140	156	156	170	175	179	184	192	195
141	153	152	163	166	175	179	184	194
141	154	153	170	169	178	183	191	197
131	148	150	158	161	178	175	189	187
127	138	142	148	156	167	168	186	182
186	223	228	256	263	278	284	310	314
150	167	169	189	190	207	210	229	241
133	147	144	156	155	170	166	181	174
119	136	139	143	145	158	162	168	170
142	156	154	170	171	179	179	180	182
152	167	169	182	183	195	194	206	210
142	156	162	168	168	180	190	195	195
139	151	154	162	161	174	182	187	193

Weight at Behavior Start
318
488
451
290
314
455
276
592
530
454
297
316
500
490
304
283
546
458
269
306
338
313
314
534
345
603
504
613
267
334
547
535
330
325
446
478
297
319
409
422
263
263
477
447
249
489

268
291
339
285
400
273
277
513
267
269
473
259
305
426
449
411
510
265
255
447
296
483
244
231
240
426
371
374
388
467
297
277
467
515
500
295
248
414
414
462
438
416
271
257
270
303
430

463
259
261
265
248
268
258
485
367
233
236
235
298
259
257

Animal ID	Sex	Mother	Treatment	Condition	BEC
101	F	6	AIE	VEx	247.3
104	M	6	Con	Stat	N/A
105	M	6	Con	VEx	N/A
107	F	6	Con	Stat	N/A
108	F	6	Con	VEx	N/A
109	M	6	AIE	VEx	270.6
113	F	91	AIE	Stat	297.7
114	M	91	Con	Stat	N/A
116	M	91	Con	VEx	N/A
118	M	91	AIE	VEx	211
121	F	91	Con	VEx	N/A
123	F	91	Con	Stat	N/A
124	M	81	AIE	Stat	164.9
127	M	81	Con	VEx	N/A
128	F	81	Con	Stat	N/A
129	F	81	Con	VEx	N/A
131	M	81	AIE	Stat	142.9
132	M	81	AIE	VEx	230.1
134	F	81	AIE	VEx	176.2
137	F	79	Con	Stat	N/A
138	F	79	Con	VEx	N/A
139	F	79	AIE	Stat	148.7
140	F	79	AIE	VEx	203.2
141	M	79	Con	VEx	N/A
144	F	79	Con	Stat	N/A
145	M	79	AIE	VEx	210.4
146	M	79	AIE	Stat	197.7
148	M	69	Con	VEx	N/A
149	M	69	Con	Stat	N/A
151	F	69	AIE	VEx	199.7
153	F	69	AIE	Stat	196.8
154	M	69	AIE	Stat	171.3
156	M	69	AIE	VEx	209.6
157	F	69	Con	VEx	N/A
158	F	69	Con	Stat	N/A
160	M	93	AIE	VEx	230.7
165	M	93	Con	VEx	N/A
167	F	93	AIE	VEx	268.5
168	F	93	AIE	Stat	245.5
172	M	83	AIE	Stat	273
173	M	83	AIE	VEx	329.4
174	F	83	AIE	VEx	238.1
175	F	83	AIE	Stat	226
177	M	83	Con	VEx	N/A
178	M	83	Con	Stat	N/A
180	F	83	Con	Stat	N/A

185	M	82	AIE	Stat	249.3
186	F	82	AIE	VEx	250.5
187	F	82	AIE	Stat	236.3
191	M	82	Con	Stat	N/A
192	F	82	Con	Stat	N/A
197	M	1	AIE	VEx	212.3
198	F	1	AIE	VEx	235.1
199	F	1	AIE	Stat	264.9
201	M	1	Con	Stat	N/A
205	F	1	Con	VEx	N/A
206	F	1	Con	Stat	N/A
212	M	2	AIE	Stat	259.5
213	F	2	Con	Stat	N/A
214	F	2	Con	VEx	N/A
216	M	2	Con	Stat	N/A
217	M	2	Con	VEx	N/A
220	M	2	AIE	VEx	184.5
223	M	3	Con	Stat	N/A
226	F	3	Con	VEx	N/A
227	F	3	Con	Stat	N/A
229	M	3	AIE	Stat	266.5
230	F	4	AIE	Stat	288.7
233	M	4	Con	Stat	N/A
301	Female	12	Con	VEx	N/A
302	Female	12	Con	VEx	N/A
303	Female	12	AIE	Stat	249.2
305	Male	12	Con	VEx	N/A
306	Male	12	Con	VEx	N/A
308	Male	12	AIE	VEx	234.3
309	Male	12	AIE	VEx	196
311	Male	21	Con	Stat	N/A
312	Female	21	Con	VEx	N/A
313	Female	21	Con	Stat	N/A
314	Male	21	Con	Stat	N/A
316	Male	21	AIE	VEx	252
318	Female	21	Con	VEx	N/A
322	Female	24	AIE	Stat	246.7
339	Male	24	Con	Stat	N/A
340	Male	24	Con	VEx	N/A
345	Male	24	Con	Stat	N/A
347	Male	21	AIE	VEx	231.7
348	Male	24	AIE	VEx	269.9
349	Female	24	Con	VEx	N/A
350	Female	21	Con	VEx	N/A
351	Female	21	AIE	VEx	231.4
352	Female	24	AIE	VEx	280.2
361	Male	24	Con	Stat	N/A

362	Male	24	AIE	Stat	327.5
363	Female	24	AIE	Stat	261.9
364	Female	21	AIE	Stat	232.5
365	Female	21	Con	Stat	N/A
366	Female	24	Con	Stat	N/A
379	Female	53	AIE	VEx	251.9
380	Female	54	AIE	VEx	225.3
383	Male	53	AIE	VEx	319.2
384	Male	54	AIE	VEx	235.1
387	Female	54	AIE	Stat	247.4
388	Female	54	AIE	Stat	258.4
389	Female	54	Con	VEx	N/A
390	Female	53	Con	VEx	N/A
391	Female	54	Con	Stat	N/A
392	Female	54	Con	Stat	N/A

Animal ID	Sex	Mother	Treatment	Condition	Distance (m)
101	F	6	AIE	VEx	480025.8286
104	M	6	Con	Stat	N/A
105	M	6	Con	VEx	302028.6857
107	F	6	Con	Stat	N/A
108	F	6	Con	VEx	480025.8286
109	M	6	AIE	VEx	302028.6857
113	F	91	AIE	Stat	N/A
114	M	91	Con	Stat	N/A
116	M	91	Con	VEx	335206.3
118	M	91	AIE	VEx	465272.5
121	F	91	Con	VEx	546260
123	F	91	Con	Stat	N/A
124	M	81	AIE	Stat	N/A
127	M	81	Con	VEx	335206.3
128	F	81	Con	Stat	N/A
129	F	81	Con	VEx	546260
131	M	81	AIE	Stat	N/A
132	M	81	AIE	VEx	465272.5
134	F	81	AIE	VEx	511456
137	F	79	Con	Stat	N/A
138	F	79	Con	VEx	419195.7
139	F	79	AIE	Stat	N/A
140	F	79	AIE	VEx	527967
141	M	79	Con	VEx	200101
144	F	79	Con	Stat	N/A
145	M	79	AIE	VEx	69474.9
146	M	79	AIE	Stat	N/A
148	M	69	Con	VEx	200101
149	M	69	Con	Stat	N/A
151	F	69	AIE	VEx	527967
153	F	69	AIE	Stat	N/A
154	M	69	AIE	Stat	N/A
156	M	69	AIE	VEx	69474.9
157	F	69	Con	VEx	419195.7
158	F	69	Con	Stat	N/A
160	M	93	AIE	VEx	261944.1
165	M	93	Con	VEx	77845.9
167	F	93	AIE	VEx	481020.1
168	F	93	AIE	Stat	N/A
172	M	83	AIE	Stat	N/A
173	M	83	AIE	VEx	261944.1
174	F	83	AIE	VEx	481020.1
175	F	83	AIE	Stat	N/A
177	M	83	Con	VEx	295942.9
178	M	83	Con	Stat	N/A
180	F	83	Con	Stat	N/A

185	M	82	AIE	Stat	N/A
186	F	82	AIE	VEx	510551.8
187	F	82	AIE	Stat	N/A
191	M	82	Con	Stat	N/A
192	F	82	Con	Stat	N/A
197	M	1	AIE	VEx	373159.6
198	F	1	AIE	VEx	448004.7
199	F	1	AIE	Stat	N/A
201	M	1	Con	Stat	N/A
205	F	1	Con	VEx	413975.1
206	F	1	Con	Stat	N/A
212	M	2	AIE	Stat	N/A
213	F	2	Con	Stat	N/A
214	F	2	Con	VEx	413975.1
216	M	2	Con	Stat	N/A
217	M	2	Con	VEx	253695.2
220	M	2	AIE	VEx	373159.6
223	M	3	Con	Stat	N/A
226	F	3	Con	VEx	448004.7
227	F	3	Con	Stat	N/A
229	M	3	AIE	Stat	N/A
230	F	4	AIE	Stat	N/A
233	M	4	Con	Stat	N/A
301	Female	12	Con	VEx	430316.7
302	Female	12	Con	VEx	430316.7
303	Female	12	AIE	Stat	N/A
305	Male	12	Con	VEx	411481.4
306	Male	12	Con	VEx	411481.4
308	Male	12	AIE	VEx	365181.3
309	Male	12	AIE	VEx	365181.3
311	Male	21	Con	Stat	N/A
312	Female	21	Con	VEx	209112.2
313	Female	21	Con	Stat	N/A
314	Male	21	Con	Stat	N/A
316	Male	21	AIE	VEx	134764.3
318	Female	21	Con	VEx	209112.2
322	Female	24	AIE	Stat	N/A
339	Male	24	Con	Stat	N/A
340	Male	24	Con	VEx	391292
345	Male	24	Con	Stat	N/A
347	Male	21	AIE	VEx	213624.4
348	Male	24	AIE	VEx	213624.4
349	Female	24	Con	VEx	253139.7
350	Female	21	Con	VEx	253139.7
351	Female	21	AIE	VEx	233209.9
352	Female	24	AIE	VEx	233209.9
361	Male	24	Con	Stat	N/A

362	Male	24	AIE	Stat	N/A
363	Female	24	AIE	Stat	N/A
364	Female	21	AIE	Stat	N/A
365	Female	21	Con	Stat	N/A
366	Female	24	Con	Stat	N/A
379	Female	53	AIE	VEx	435714.4
380	Female	54	AIE	VEx	435714.4
383	Male	53	AIE	VEx	320853.5
384	Male	54	AIE	VEx	320853.5
387	Female	54	AIE	Stat	N/A
388	Female	54	AIE	Stat	N/A
389	Female	54	Con	VEx	556223.8
390	Female	53	Con	VEx	556223.8
391	Female	54	Con	Stat	N/A
392	Female	54	Con	Stat	N/A

Animal ID	Treatment	Condition	Sex	AcqTrials	AcqOmissi	AcqTC	AcqErrors	AcqLatenc	AcqLatenc
114	Con	Stat	M	83	0	83	21	0.48	0.89
149	Con	Stat	M	87	0	87	27	0.44	0.66
178	Con	Stat	M	46	0	46	12	0.83	0.44
201	Con	Stat	M	74	0	74	26	1.25	1.13
216	Con	Stat	M	69	0	69	21	0.9	0.76
223	Con	Stat	M	80	1	79	24	1.86	0.55
233	Con	Stat	M	78	0	78	25	0.67	1.43
311	Con	Stat	M	91	4	87	30	1.95	1.15
345	Con	Stat	M	104	0	104	41	0.7	0.66
104	Con	Stat	M	51	0	51	23	1.71	0.73
314	Con	Stat	M	84	0	84	32	0.5	1.36
339	Con	Stat	M	82	0	82	19	1.29	1.03
361	Con	Stat	M	67	0	67	30	0.36	1.03
116	Con	VEx	M	82	0	82	23	0.95	1.93
217	Con	VEx	M	56	0	56	25	0.77	0.6
305	Con	VEx	M	90	0	90	26	0.49	0.5
306	Con	VEx	M	83	1	82	32	0.88	0.57
105	Con	VEx	M	93	0	93	42	0.89	0.68
127	Con	VEx	M	138	1	137	55	0.61	0.6
141	Con	VEx	M	69	0	69	25	0.45	0.79
148	Con	VEx	M	102	0	102	42	0.52	0.75
340	Con	VEx	M	72	0	72	26	0.95	0.63
131	AIE	Stat	M	77	0	77	19	0.54	0.63
154	AIE	Stat	M	135	0	135	40	0.4	0.4
172	AIE	Stat	M	81	0	81	33	1.88	1.01
212	AIE	Stat	M	136	0	136	44	0.58	1.22
229	AIE	Stat	M	65	0	65	20	1.41	0.78
362	AIE	Stat	M	50	0	50	18	0.43	0.61
124	AIE	Stat	M	48	0	48	13	0.94	0.73
146	AIE	Stat	M	54	0	54	29	0.42	0.84
185	AIE	Stat	M	128	0	128	49	0.28	0.77
118	AIE	VEx	M	112	0	112	37	0.73	0.59
132	AIE	VEx	M	94	0	94	31	0.38	0.51
145	AIE	VEx	M	30	0	30	5	0.44	
160	AIE	VEx	M	35	0	35	6	0.33	0.61
197	AIE	VEx	M	80	0	80	40	0.65	0.66
308	AIE	VEx	M	101	0	101	34	0.79	0.46
309	AIE	VEx	M	47	0	47	12	0.81	0.48
316	AIE	VEx	M	43	0	43	16	0.71	0.79
383	AIE	VEx	M	59	0	59	21	0.9	0.69
109	AIE	VEx	M	57	0	57	11	0.8	0.8
156	AIE	VEx	M	97	0	97	27	0.3	0.66
173	AIE	VEx	M	83	0	83	26	0.66	0.72
220	AIE	VEx	M	59	0	59	31	0.78	0.58
348	AIE	VEx	M	82	0	82	39	0.85	0.57
384	AIE	VEx	M	49	0	49	17	0.61	0.84

347	AIE	VEx	M	112	1	111	34	0.68	0.5
123	Con	Stat	F	77	0	77	29	0.76	0.68
128	Con	Stat	F	63	0	63	19	0.79	0.74
137	Con	Stat	F	90	0	90	20	0.95	0.71
144	Con	Stat	F	58	0	58	21	1.24	0.62
192	Con	Stat	F	100	0	100	32	0.29	0.52
213	Con	Stat	F	31	0	31	9	1.44	
227	Con	Stat	F	35	1	34	12	1.22	0.76
313	Con	Stat	F	67	0	67	22	1.83	1.23
365	Con	Stat	F	56	1	55	21	0.84	1.03
107	Con	Stat	F	86	4	82	20	2.02	0.97
158	Con	Stat	F	30	0	30	10	0.54	
162	Con	Stat	F	75	0	75	31	1.09	0.46
180	Con	Stat	F	74	1	73	26	1.45	0.4
206	Con	Stat	F	75	0	75	20	0.95	0.78
366	Con	Stat	F	58	0	58	19	0.71	0.6
391	Con	Stat	F	88	0	88	25	2.7	0.95
392	Con	Stat	F	50	0	50	16	0.97	0.63
214	Con	VEx	F	103	0	103	47	1.18	0.76
312	Con	VEx	F	69	1	68	20	1.48	0.62
350	Con	VEx	F	99	0	99	25	1.28	0.72
389	Con	VEx	F	41	0	41	18	0.68	0.74
108	Con	VEx	F	71	1	70	26	0.83	0.76
121	Con	VEx	F	86	0	86	37	0.62	0.66
129	Con	VEx	F	69	0	69	21	1.72	0.87
138	Con	VEx	F	73	0	73	29	0.5	0.62
157	Con	VEx	F	109	0	109	44	0.93	1
177	Con	VEx	F	65	0	65	19	0.79	0.55
205	Con	VEx	F	56	0	56	21	2.36	0.92
226	Con	VEx	F	90	0	90	31	1.03	0.73
301	Con	VEx	F	134	0	134	41	0.91	0.65
302	Con	VEx	F	61	2	59	18	0.73	0.77
318	Con	VEx	F	38	1	37	14	1.07	0.84
389	Con	VEx	F	41	0	41	18	0.68	0.74
390	Con	VEx	F	76	0	76	24	1.57	0.63
113	AIE	Stat	F	135	0	135	48	0.63	0.67
139	AIE	Stat	F	78	2	76	26	1.02	0.86
168	AIE	Stat	F	30	0	30	9	1.15	
175	AIE	Stat	F	74	0	74	31	1.13	0.58
187	AIE	Stat	F	35	0	35	5	1.25	0.7
199	AIE	Stat	F	47	0	47	18	1.04	0.77
230	AIE	Stat	F	55	0	55	18	1.62	0.98
322	AIE	Stat	F	46	0	46	6	1.38	0.74
364	AIE	Stat	F	71	0	71	22	1.8	0.67
387	AIE	Stat	F	71	0	71	30	1.69	0.69
153	AIE	Stat	F	74	0	74	28	1.2	0.74
303	AIE	Stat	F	37	0	37	10	1.46	0.61

363	AIE	Stat	F	53	0	53	12	1.05	1.88
388	AIE	Stat	F	77	0	77	23	1.09	0.84
101	AIE	VEx	F	67	0	67	14	0.74	0.72
134	AIE	VEx	F	64	0	64	28	1.39	0.96
140	AIE	VEx	F	81	0	81	26	0.6	0.63
167	AIE	VEx	F	128	0	128	46	0.61	0.68
198	AIE	VEx	F	103	2	101	38	1.48	0.73
349	AIE	VEx	F	53	3	50	16	2.79	1.05
379	AIE	VEx	F	45	0	45	13	1.15	0.84
380	AIE	VEx	F	54	2	52	16	1.06	0.75
151	AIE	VEx	F	52	0	52	13	1.65	0.87
186	AIE	VEx	F	89	0	89	29	0.91	0.93
351	AIE	VEx	F	46	2	44	21	1.98	0.59
352	AIE	VEx	F	57	0	57	20	2.22	0.56

SS1trials	SS1Omissio	SS1TC	SS1TCafter	SS1Errors	SS1Errorsa	SS1Latency	SS1Latency	SS1Latency	SS1Latency
250	0	250	230	62	62	0.34	0.31	1	1
178	0	178	158	53	53	0.28	0.28	0.67	0.68
127	0	127	107	38	38	0.62	0.55	0.48	0.48
71	0	71	51	14	14	0.37	0.52	0.93	0.93
301	1	300	280	114	114	0.93	0.94	0.84	0.86
74	0	74	54	14	14	1.4	1.52	0.57	0.58
170	0	170	150	54	54	0.47	0.44	0.97	0.94
269	0	269	249	112	112	1.05	1.08	0.78	0.77
50	0	50	30	6	6	0.76	0.78	0.6	0.63
194	0	194	174	61	61	1.19	1.16	0.68	0.68
113	0	113	93	19	19	0.43	0.42	1.15	1.11
153	0	153	133	43	43	1.49	1.49	0.94	0.94
77	0	77	57	11	11	0.38	0.37	1.11	1.13
85	0	85	65	13	13	0.62	0.52	0.94	0.86
150	0	150	130	38	38	0.9	0.92	0.58	0.58
106	0	106	86	28	28	0.6	0.61	0.5	0.5
159	0	159	139	49	49	0.81	0.69	0.73	0.73
76	0	76	56	19	19	1.09	1.13	0.69	0.7
352	0	352	332	143	143	0.38	0.36	0.79	0.77
546	0	546	526	204	204	0.37	0.37	0.54	0.54
274	0	274	254	97	97	0.43	0.42	0.93	0.9
95	0	95	75	16	16	0.77	0.74	0.56	0.55
53	0	53	33	5	5	0.6	0.57	0.63	0.6
252	1	251	231	86	86	0.59	0.61	0.47	0.47
178	1	177	157	66	66	1.93	2.01	1.04	1.03
450	0	450	430	185	185	0.77	0.77	1.1	1.1
124	2	122	102	39	39	1.59	1.67	0.92	0.94
94	0	94	74	20	20	0.57	0.54	0.93	0.98
261	0	261	241	96	96	0.76	0.78	0.74	0.74
194	0	194	174	52	52	0.4	0.37	0.51	0.51
250	0	250	230	72	72	0.31	0.3	0.64	0.63
89	0	89	69	12	12	0.47	0.45	0.65	0.69
253	0	253	233	83	83	0.33	0.33	0.52	0.52
295	0	295	275	80	80	0.34	0.34	0.39	0.38
201	0	201	181	61	61	0.56	0.58	0.59	0.56
94	0	94	74	23	23	0.78	0.84	0.62	0.63
75	0	75	55	19	19	0.49	0.41	0.73	0.79
58	0	58	38	6	6	0.74	0.87	0.52	0.52
132	7	125	105	30	30	2.73	2.62	0.78	0.78
114	0	114	94	27	27	0.68	0.67	0.78	0.79
72	0	72	52	16	16	1.07	1.07	0.78	0.84
264	0	264	244	72	72	0.3	0.31	0.68	0.67
250	0	250	230	103	103	0.28	0.26	1.11	1.15
86	0	86	66	19	19	0.83	0.91	0.63	0.66
122	0	122	102	41	41	1.27	1.35	0.57	0.57
354	0	354	334	142	142	0.49	0.48	0.91	0.91

135	0	135	115	38	38	0.51	0.51	0.61	0.63
121	0	121	101	27	27	0.35	0.33	0.8	0.81
50	1	49	29	4	4	1.2	1.53	0.9	0.94
130	0	130	110	45	45	0.78	0.77	0.91	0.92
315	0	315	295	83	83	1.01	1.03	0.62	0.62
254	0	254	234	94	94	0.34	0.34	0.51	0.52
50	0	50	30	1	1	1.07	1.12	0.81	0.81
217	3	214	194	55	55	1.33	1.31	0.72	0.72
650	10	640	620	273	273	1.81	1.77	0.97	0.97
141	3	138	118	35	35	1.72	1.74	1.14	1.15
650	15	635	615	284	284	1.86	1.84	0.99	1
84	0	84	64	8	8	0.41	0.36	0.77	0.79
313	5	308	288	108	108	1.06	1.03	0.5	0.51
188	0	188	168	44	44	1.5	1.46	0.44	0.44
182	1	181	161	54	54	0.92	0.89	0.84	0.83
94	1	93	73	23	23	1.58	1.85	0.62	0.62
151	3	148	128	48	48	2.3	2.36	1.04	1.06
141	3	138	118	35	35	1.18	1.24	0.83	0.86
197	0	197	177	64	64	1.07	1.07	0.75	0.75
352	0	352	332	151	151	1.44	1.4	0.65	0.65
450	0	450	430	188	188	1.02	1.01	0.69	0.69
135	0	135	115	31	31	0.39	0.39	0.73	0.73
90	0	90	70	17	17	1.18	1.22	0.8	0.82
250	0	250	230	83	83	0.56	0.55	0.55	0.55
365	3	362	342	132	132	0.8	0.74	0.74	0.73
145	0	145	125	42	42	0.55	0.58	0.51	0.5
270	0	270	250	110	110	0.43	0.4	0.67	0.67
257	0	257	237	76	76	0.76	0.78	0.59	0.59
294	7	287	267	100	100	1.45	1.42	0.82	0.83
106	0	106	86	29	29	0.9	0.85	0.56	0.54
128	0	128	108	28	28	0.66	0.68	0.62	0.61
119	0	119	99	27	27	0.79	0.86	0.82	0.85
61	0	61	41	7	7	0.84	0.82	0.76	0.72
135	0	135	115	31	31	0.39	0.39	0.73	0.73
93	0	93	73	21	21	1.05	1.14	0.87	0.68
474	0	474	454	180	180	0.61	0.58	0.56	0.56
142	1	141	121	43	43	1.03	1.03	0.68	0.7
131	0	131	111	40	40	0.6	0.56	0.73	0.7
314	0	314	294	131	131	0.95	0.96	0.57	0.57
137	0	137	117	35	35	1.22	1.17	0.78	0.77
203	0	203	183	64	64	0.84	0.83	0.68	0.68
138	1	137	117	48	48	1.63	1.66	0.88	0.89
541	9	532	512	240	240	1.58	1.57	0.7	0.7
277	0	277	257	115	115	1.1	1.02	0.87	0.89
349	0	349	329	111	111	1.48	1.48	0.76	0.77
250	0	250	230	101	101	1.12	1.13	0.6	0.59
168	0	168	148	44	44	1.09	1.03	0.54	0.55

137	0	137	117	46	46	1.77	1.81	0.98	0.87
91	0	91	71	28	28	1.06	1.13	1.01	1.05
310	2	308	288	117	117	0.82	0.84	0.67	0.66
148	0	148	128	54	54	1.03	1.01	0.83	0.85
205	0	205	185	72	72	0.42	0.42	0.64	0.61
120	0	120	100	39	39	0.62	0.66	0.82	0.86
211	4	207	187	66	66	1.81	1.79	0.99	1.04
319	4	315	295	121	121	2.16	2.09	1.05	1.08
92	1	91	71	23	23	1.19	1.22	0.84	0.81
277	7	270	250	89	89	1.34	1.37	0.8	0.81
117	0	117	97	27	27	1.13	1.13	0.88	0.92
250	1	249	229	73	73	0.81	0.77	0.96	0.97
339	11	328	308	141	141	1.67	1.68	0.64	0.64
141	0	141	121	31	31	1.36	1.34	0.63	0.65

SS1Persev	SS1Persev	SS1Regres	SS1Regres	SS2Trials	SS2Omissi	SS2TC	SS2TCafter	SS2Errors	SS2Errorsa
11	0.18	51	0.82	59	0	59	39	11	11
24	0.45	29	0.55	115	0	115	95	30	30
19	0.5	19	0.5	91	0	91	71	29	29
11	0.79	3	0.21	86	1	85	65	29	29
97	0.85	17	0.15	143	2	141	121	43	43
0	0	14	1	77	0	77	57	16	16
30	0.56	24	0.44	68	0	68	48	14	14
112	1	0	0	92	0	92	72	43	43
0	0	6	1	147	1	146	126	42	42
18	0.3	43	0.7	88	0	88	68	24	24
0	0	19	1	99	0	99	79	30	30
14	0.33	29	0.67	54	0	54	34	7	7
0	0	11	1	83	0	83	63	16	16
8	0.62	5	0.38	69	0	69	49	10	10
17	0.45	21	0.55	75	0	75	55	13	13
0	0	28	1	127	0	127	107	35	35
40	0.82	9	0.18	72	0	72	52	10	10
19	1	0	0	141	0	141	121	50	50
142	0.99	1	0.01	74	0	74	54	17	17
131	0.64	73	0.36	65	0	65	45	15	15
76	0.78	21	0.22	72	0	72	52	14	14
0	0	16	1	169	0	169	149	40	40
0	0	5	1	86	0	86	66	17	17
69	0.8	17	0.2	93	0	93	73	25	25
64	0.97	2	0.03	82	0	82	62	21	21
96	0.52	89	0.48	90	0	90	70	19	19
39	1	0	0	68	0	68	48	21	21
0	0	20	1	124	1	123	103	40	40
86	0.9	10	0.1	59	0	59	39	12	12
0	0	52	1	84	0	84	64	26	26
0	0	72	1	155	0	155	135	49	49
0	0	12	1	140	0	140	120	49	49
11	0.13	72	0.87	74	0	74	54	21	21
13	0.16	67	0.84	115	0	115	95	30	30
21	0.34	40	0.66	51	0	51	31	12	12
22	0.96	1	0.04	67	0	67	47	15	15
19	1	0	0	111	0	111	91	32	32
0	0	6	1	89	0	89	69	24	24
11	0.37	19	0.63	94	0	94	74	30	30
17	0.63	10	0.37	76	0	76	56	23	23
16	1	0	0	92	0	92	72	23	23
11	0.15	61	0.85	103	0	103	83	37	37
96	0.93	7	0.07	85	0	85	65	26	26
0	0	19	1	53	0	53	33	10	10
41	1	0	0	89	6	83	63	32	32
31	0.22	111	0.78	102	0	102	82	27	27

12	0.32	26	0.68	115	0	115	95	22	22
0	0	27	1	125	0	125	105	41	41
0	0	4	1	105	2	103	83	25	25
27	0.6	18	0.4	79	0	79	59	21	21
10	0.12	73	0.88	92	0	92	72	16	16
91	0.97	3	0.03	104	0	104	84	28	28
0	0	1	1	104	0	104	84	35	35
0	0	55	1	68	0	68	48	17	17
253	0.93	20	0.07	55	0	55	35	13	13
17	0.49	18	0.51	99	13	86	66	33	33
196	0.69	88	0.31	65	2	63	43	11	11
0	0	8	1	116	0	116	96	38	38
78	0.72	30	0.28	60	0	60	40	14	14
0	0	44	1	141	5	136	116	39	39
25	0.46	29	0.54	80	0	80	60	19	19
22	0.96	1	0.04	162	7	155	135	51	51
26	0.54	22	0.46	94	1	93	73	25	25
0	0	35	1	89	1	88	68	30	30
0	0	64	1	79	0	79	59	24	24
147	0.97	4	0.03	100	0	100	80	33	33
186	0.99	2	0.01	62	0	62	42	16	16
0	0	31	1	106	0	106	86	21	21
10	0.59	7	0.41	106	0	106	86	27	27
55	0.66	28	0.34	77	0	77	57	21	21
87	0.66	45	0.34	67	0	67	47	15	15
18	0.43	24	0.57	123	0	123	103	35	35
105	0.95	5	0.05	77	0	77	57	25	25
38	0.5	38	0.5	72	0	72	52	18	18
59	0.59	41	0.41	75	0	75	55	18	18
19	0.66	10	0.34	77	0	77	57	20	20
0	0	28	1	99	0	99	79	33	33
0	0	27	1	59	0	59	39	8	8
0	0	7	1	96	4	92	72	33	33
0	0	31	1	106	0	106	86	21	21
0	0	21	1	101	4	97	77	37	37
28	0.16	152	0.84	63	0	63	43	15	15
42	0.98	1	0.02	55	0	55	35	11	11
12	0.3	28	0.7	174	0	174	154	78	78
131	1	0	0	81	0	81	61	22	22
9	0.26	26	0.74	93	3	90	70	26	26
41	0.64	23	0.36	68	0	68	48	16	16
43	0.9	5	0.1	50	0	50	30	8	8
142	0.59	98	0.41	52	0	52	32	8	8
115	1	0	0	57	1	56	36	9	9
16	0.14	95	0.86	77	0	77	57	16	16
100	0.99	1	0.01	89	0	89	69	22	22
19	0.43	25	0.57	98	0	98	78	20	20

46	1	0	0	80	0	80	60	15	15
28	1	0	0	96	0	96	76	26	26
48	0.41	41	0.35	72	0	72	52	17	17
51	0.94	3	0.06	72	0	72	52	19	19
0	0	72	1	88	0	88	68	24	24
39	1	0	0	133	0	133	113	45	45
0	0	66	1	167	1	166	146	69	69
107	0.88	14	0.12	118	0	118	98	38	38
13	0.57	10	0.43	66	0	66	46	19	19
52	0.58	37	0.42	79	0	79	59	23	23
0	0	27	1	78	0	78	58	27	27
48	0.66	25	0.34	71	0	71	51	22	22
112	0.79	29	0.21	99	0	99	79	43	43
11	0.35	20	0.65	107	5	102	82	38	38

SS2Latency	SS2Latency	SS2Latency	SS2Latency	SS2Perseve	SS2Perseve	SS2Regress	SS2Regress	RevTrials	RevOmissi
0.3	0.29	0.8	0.79	11	1	0	0	161	2
0.2	0.18	1.08	1.15	19	0.63	11	0.37	100	0
0.41	0.38	0.53	0.55	22	0.76	7	0.24	93	0
0.66	0.45	0.73	0.73	29	1	0	0	182	19
0.78	0.77	0.83	0.85	0	0	43	1	99	0
1.35	1.31	0.6	0.62	12	0.75	4	0.25	89	0
0.38	0.38	0.89	0.91	13	0.93	1	0.07	69	0
1	1	0.75	0.8	43	1	0	0	91	0
0.76	0.76	0.98	1.02	28	0.67	14	0.33	143	1
1.01	1.04	0.7	0.71	24	1	0	0	107	0
0.35	0.37	0.76	0.73	27	0.9	3	0.1	86	0
0.83	0.92	1	1.03	0	0	7	1	108	1
0.44	0.44	0.87	0.88	9	0.56	7	0.44	55	0
0.4	0.42	1.35	1.43	9	0.9	1	0.1	98	0
1.05	1.05	0.55	0.52	0	0	13	1	120	0
0.4	0.4	0.48	0.47	26	0.74	9	0.26	53	0
0.7	0.71	0.6	0.56	0	0	10	1	69	0
1.18	1.2	0.61	0.6	46	0.92	4	0.08	131	0
0.28	0.27	0.92	0.98	0	0	17	1	99	0
0.34	0.28	0.46	0.47	0	0	15	1	108	0
0.31	0.3	0.75	0.75	14	1	0	0	95	0
0.49	0.47	0.59	0.57	0	0	40	1	138	0
0.33	0.31	0.77	0.83	8	0.47	9	0.53	64	0
0.47	0.31	0.76	0.83	20	0.8	5	0.2	156	0
0.92	0.7	1.39	1.5	13	0.62	8	0.38	146	0
0.44	0.46	1.2	1.21	0	0	19	1	83	0
1.67	1.83	0.95	1.04	21	1	0	0	65	0
0.53	0.57	1.17	1.19	29	0.73	11	0.28	92	0
0.4	0.35	0.71	0.75	12	1	0	0	87	0
0.37	0.39	0.39	0.36	26	1	0	0	94	2
0.26	0.27	0.51	0.51	42	0.86	7	0.14	114	0
0.24	0.22	0.76	0.8	46	0.94	3	0.06	127	0
0.18	0.19	0.45	0.43	21	1	0	0	80	0
0.46	0.44	0.51	0.49	17	0.57	13	0.43	163	0
0.33	0.28	0.44	0.42	12	1	0	0	93	0
0.39	0.38	0.83	0.98	13	0.87	2	0.13	148	0
0.55	0.57	0.68	0.67	29	0.91	3	0.09	78	0
0.5	0.48	0.51	0.53	14	0.58	10	0.42	71	1
1.62	1.71	0.6	0.59	30	1	0	0	98	1
0.37	0.42	0.78	0.76	23	1	0	0	64	0
0.67	0.66	0.78	0.81	14	0.61	9	0.39	106	1
0.7	0.74	0.85	0.69	37	1	0	0	83	0
0.29	0.31	1.71	1.93	24	0.92	2	0.08	89	0
0.49	0.45	0.64	0.66	10	1	0	0	68	0
2.41	2.36	0.69	0.77	32	1	0	0	97	1
1.02	1.19	0.73	0.72	0	0	27	1	146	0

0.44	0.44	0.51	0.5	0	0	22	1	127	0
0.29	0.28	0.64	0.63	35	0.85	6	0.15	112	0
1.63	1.72	0.95	0.99	0	0	25	1	114	0
0.38	0.34	0.87	0.84	19	0.9	2	0.1	133	0
0.77	0.68	0.6	0.62	11	0.69	5	0.31	78	0
0.48	0.47	0.64	0.65	22	0.79	6	0.21	74	0
0.74	0.75	0.76	0.73	35	1	0	0	82	0
1.25	1.17	0.72	0.73	15	0.88	2	0.12	102	0
1.21	1.21	0.8	0.86	13	1	0	0	166	3
2.6	2.54	1.23	1.4	33	1	0	0	97	2
1.05	0.85	1.03	1.07	0	0	11	1	78	0
0.4	0.37	0.58	0.55	20	0.53	18	0.47	117	0
0.79	0.67	0.53	0.54	14	1	0	0	105	1
1.61	1.66	0.45	0.46	22	0.56	17	0.44	50	7
0.65	0.58	0.92	1.01	18	0.95	1	0.05	63	0
2.04	2.07	0.59	0.59	37	0.73	14	0.27	142	2
1.97	1.85	1.18	1.25	19	0.76	6	0.24	98	0
1.05	1.02	0.71	0.65	30	1	0	0	149	0
1.27	1.34	0.78	0.81	24	1	0	0	71	1
1.31	1.42	0.68	0.69	30	0.91	3	0.09	147	0
0.58	0.57	0.79	0.86	16	1	0	0	191	7
0.51	0.47	0.76	0.78	11	0.52	10	0.48	71	0
0.62	0.63	0.72	0.73	18	0.67	9	0.33	104	0
0.36	0.38	0.58	0.61	21	1	0	0	57	0
0.9	0.91	0.62	0.6	15	1	0	0	96	0
0.81	0.83	0.53	0.54	30	0.86	5	0.14	118	0
0.5	0.45	0.76	0.76	25	1	0	0	57	0
0.68	0.67	0.67	0.71	18	1	0	0	72	0
2.04	2.12	0.86	0.89	18	1	0	0	110	4
1.07	1.05	0.51	0.51	20	1	0	0	50	0
0.54	0.52	0.58	0.57	33	1	0	0	122	0
0.65	0.54	0.84	0.9	0	0	8	1	114	0
1.63	1.62	0.67	0.63	33	1	0	0	124	0
0.51	0.47	0.76	0.78	11	0.52	10	0.48	71	0
1.79	1.99	0.54	0.54	37	1	0	0	99	0
0.58	0.57	0.64	0.67	15	1	0	0	70	0
1.1	0.92	0.79	0.85	11	1	0	0	112	0
0.39	0.37	0.51	0.47	78	1	0	0	136	0
0.58	0.57	0.51	0.48	22	1	0	0	69	0
1.35	1.2	0.91	0.98	14	0.54	12	0.46	97	4
0.91	1.04	0.7	0.72	16	1	0	0	61	0
1.64	1.72	0.87	0.9	8	1	0	0	70	0
1.05	0.99	0.56	0.57	8	1	0	0	63	0
1.09	0.85	0.8	0.75	9	1	0	0	98	0
1.01	0.9	0.81	0.84	16	1	0	0	130	0
0.74	0.6	0.47	0.45	18	0.82	4	0.18	96	1
0.68	0.63	0.57	0.56	0	0	20	1	71	1

1.52	1.6	0.83	0.87	11	0.73	4	0.27	54	0
1.4	1.48	1.11	1.17	25	0.96	1	0.04	94	0
0.49	0.48	0.65	0.67	13	0.76	4	0.24	58	0
1.04	1.11	0.72	0.68	19	1	0	0	131	0
0.36	0.27	0.41	0.38	24	1	0	0	88	0
0.89	0.89	0.78	0.79	42	0.93	3	0.07	118	1
1.84	1.9	1.28	1.29	67	0.97	2	0.03	104	0
1.48	1.62	1.04	1.01	20	0.53	18	0.47	91	0
0.89	0.99	0.78	0.77	19	1	0	0	88	0
0.64	0.64	0.6	0.59	23	1	0	0	90	0
1.05	0.98	1.01	1.1	27	1	0	0	82	0
0.5	0.42	1.28	1.38	22	1	0	0	116	0
1.74	1.91	0.59	0.61	43	1	0	0	71	0
1.87	2.05	0.94	1.08	38	1	0	0	87	1

RevTC	RevTCafter	RevErrors	RevErrorsa	RevLatency	RevLatency	RevLatency	RevLatency	RevPersev	RevPersev
159	139	66	66	0.71	0.76	0.67	0.69	31	0.47
100	80	33	33	0.24	0.24	0.72	0.68	17	0.52
93	73	33	33	0.31	0.29	0.42	0.36	19	0.58
163	143	90	90	0.47	0.53	1.08	0.83	44	0.49
99	79	39	39	0.56	0.51	0.75	0.75	33	0.85
89	69	34	34	1.26	1.3	0.54	0.52	22	0.65
69	49	27	27	0.3	0.27	1.02	1.07	23	0.85
91	71	34	34	1.07	1.11	0.69	0.66	31	0.91
142	122	50	50	0.47	0.42	0.55	0.51	0	0
107	87	35	35	1.29	1.33	0.71	0.71	18	0.51
86	66	32	32	0.59	0.66	0.96	1.05	19	0.59
107	87	32	32	0.52	0.51	0.76	0.73	0	0
55	35	18	18	0.35	0.27	0.95	1.06	18	1
98	78	34	34	0.78	0.82	0.94	0.6	21	0.62
120	100	41	41	0.89	0.9	0.47	0.48	25	0.61
53	33	13	13	0.32	0.3	0.45	0.44	13	1
69	49	19	19	0.71	0.74	0.51	0.52	16	0.84
131	111	65	65	1.15	1.19	0.63	0.65	48	0.74
99	79	37	37	0.2	0.2	0.83	0.57	0	0
108	88	47	47	0.26	0.26	0.49	0.52	32	0.68
95	75	39	39	0.27	0.27	0.64	0.62	29	0.74
138	118	66	66	0.49	0.5	0.69	0.72	64	0.97
64	44	22	22	0.36	0.28	0.63	0.64	20	0.91
156	136	76	76	0.78	0.87	0.43	0.37	0	0
146	126	61	61	0.79	0.79	1.03	1	34	0.56
83	63	31	31	0.47	0.5	1	1.01	0	0
65	45	21	21	0.7	0.63	0.73	0.67	18	0.86
92	72	31	31	0.44	0.43	0.9	0.92	17	0.55
87	67	25	25	0.36	0.39	0.8	0.82	19	0.76
92	72	37	37	0.38	0.39	0.51	0.5	31	0.84
114	94	55	55	0.24	0.24	0.61	0.63	48	0.87
127	107	52	52	0.2	0.2	0.64	0.63	44	0.85
80	60	21	21	0.16	0.16	0.35	0.35	0	0
163	143	67	67	0.7	0.74	0.44	0.44	0	0
93	73	42	42	0.37	0.41	0.54	0.57	35	0.83
148	128	55	55	0.44	0.46	0.53	0.48	43	0.78
78	58	23	23	0.56	0.48	0.67	0.67	18	0.78
70	50	27	27	0.53	0.57	0.46	0.44	23	0.85
97	77	24	24	1.85	1.9	0.74	0.73	0	0
64	44	15	15	0.66	0.56	0.88	0.81	14	0.93
105	85	41	41	0.56	0.54	0.69	0.68	22	0.54
83	63	25	25	0.45	0.47	0.61	0.51	16	0.64
89	69	32	32	0.23	0.24	1.29	1.26	0	0
68	48	16	16	0.48	0.49	0.67	0.66	15	0.94
96	76	35	35	2.49	2.49	0.64	0.63	0	0
146	126	66	66	0.83	0.86	0.83	0.86	54	0.82

127	107	47	47	0.33	0.34	0.64	0.68	37	0.79
112	92	40	40	0.24	0.22	0.82	0.88	0	0
114	94	36	36	1.2	1.17	0.8	0.76	28	0.78
133	113	58	58	0.53	0.57	0.58	0.55	20	0.34
78	58	31	31	0.63	0.68	0.47	0.49	30	0.97
74	54	26	26	0.7	0.73	0.55	0.44	22	0.85
82	62	29	29	0.53	0.4	0.76	0.78	20	0.69
102	82	38	38	1.26	1.1	0.72	0.74	18	0.47
163	143	48	48	1.59	1.63	0.82	0.83	16	0.33
95	75	35	35	2.47	2.24	1.06	1.03	19	0.54
78	58	26	26	0.99	0.79	1.09	0.96	18	0.69
117	97	38	38	0.46	0.41	0.65	0.67	22	0.58
104	84	34	34	0.47	0.49	0.44	0.4	0	0
43	23	11	11	2.2	2.19	0.45	0.44	11	1
63	43	14	14	0.35	0.37	0.96	0.94	13	0.93
140	120	60	60	1.64	1.62	0.5	0.5	44	0.73
98	78	44	44	2.18	2.18	0.99	0.93	31	0.7
149	129	53	53	1.08	1.07	0.54	0.52	37	0.7
70	50	20	20	1.17	1.29	0.76	0.79	18	0.9
147	127	43	43	0.91	0.89	0.56	0.56	0	0
184	164	71	71	0.87	0.9	0.68	0.66	19	0.27
71	51	29	29	0.35	0.3	1.03	1.24	29	1
104	84	33	33	0.59	0.59	0.75	0.76	29	0.88
57	37	14	14	0.7	0.78	0.53	0.52	14	1
96	76	24	24	0.18	0.16	0.53	0.59	0	0
118	98	39	39	0.6	0.56	0.44	0.43	18	0.46
57	37	15	15	0.64	0.61	0.62	0.62	15	1
72	52	30	30	0.86	0.87	0.56	0.54	30	1
106	86	23	23	2.15	2.34	0.72	0.69	17	0.74
50	30	9	9	0.91	0.8	0.92	1.19	0	0
122	102	61	61	0.56	0.55	0.55	0.54	51	0.84
114	94	51	51	0.55	0.5	0.68	0.63	39	0.76
124	104	47	47	0.69	0.63	0.66	0.68	0	0
71	51	29	29	0.35	0.3	1.03	1.24	29	1
99	79	34	34	1.46	1.65	0.56	0.57	0	0
70	50	23	23	0.34	0.33	0.51	0.48	19	0.83
112	92	37	37	0.9	0.99	0.64	0.63	0	0
136	116	50	50	0.27	0.25	0.75	0.78	0	0
69	49	24	24	0.35	0.34	0.6	0.64	22	0.92
93	73	30	30	1.19	1.27	0.77	0.7	0	0
61	41	20	20	0.8	0.76	0.72	0.71	20	1
70	50	19	19	1.86	1.77	0.81	0.85	17	0.89
63	43	16	16	1.28	1.3	0.54	0.58	0	0
98	78	47	47	0.9	0.89	0.78	0.83	45	0.96
130	110	61	61	1.2	1.27	0.73	0.7	49	0.8
95	75	37	37	1.02	0.99	0.53	0.58	31	0.84
70	50	24	24	1.38	1.34	0.86	1.01	18	0.75

54	34	11	11	1.82	1.82	0.82	0.83	0	0
94	74	48	48	1.06	1.06	0.88	0.84	47	0.98
58	38	19	19	0.49	0.54	0.63	0.64	19	1
131	111	39	39	1.1	1.16	0.93	0.94	19	0.49
88	68	36	36	0.22	0.22	0.39	0.4	36	1
117	97	56	56	0.76	0.72	0.61	0.58	45	0.8
104	84	47	47	1.37	1.51	0.85	0.8	36	0.77
91	71	31	31	1.76	1.84	0.88	0.91	27	0.87
88	68	25	25	1.42	1.41	0.86	0.91	0	0
90	70	39	39	0.65	0.66	0.64	0.68	39	1
82	62	17	17	1.12	0.96	0.85	0.79	0	0
116	96	40	40	0.32	0.3	0.98	0.87	32	0.8
71	51	22	22	1.71	1.89	0.54	0.56	20	0.91
86	66	27	27	1.46	1.56	0.56	0.55	0	0

RevRegres	RevRegressive
35	0.53
16	0.48
14	0.42
46	0.51
6	0.15
12	0.35
4	0.15
3	0.09
50	1
17	0.49
13	0.41
32	1
0	0
13	0.38
16	0.39
0	0
3	0.16
17	0.26
37	1
15	0.32
10	0.26
2	0.03
2	0.09
76	1
27	0.44
31	1
3	0.14
14	0.45
6	0.24
6	0.16
7	0.13
8	0.15
21	1
67	1
7	0.17
12	0.22
5	0.22
4	0.15
24	1
1	0.07
19	0.46
9	0.36
32	1
1	0.06
35	1
12	0.18

10	0.21
40	1
8	0.22
38	0.66
1	0.03
4	0.15
9	0.31
20	0.53
32	0.67
16	0.46
8	0.31
16	0.42
34	1
0	0
1	0.07
16	0.27
13	0.3
16	0.3
2	0.1
43	1
52	0.73
0	0
4	0.12
0	0
24	1
21	0.54
0	0
0	0
6	0.26
9	1
10	0.16
12	0.24
47	1
0	0
34	1
4	0.17
37	1
50	1
2	0.08
30	1
0	0
2	0.11
16	1
2	0.04
12	0.2
6	0.16
6	0.25

11	1
1	0.02
0	0
20	0.51
0	0
11	0.2
11	0.23
4	0.13
25	1
0	0
17	1
8	0.2
2	0.09
27	1

Animal ID	Sex	Treatment	Condition	Litter	SA Score	Arm Entries
123	F	Con	Stat	91	22.2	48
128	F	Con	Stat	81	24.4	44
137	F	Con	Stat	79	21.7	49
144	F	Con	Stat	79	30.6	39
192	F	Con	Stat	82	24.4	44
213	F	Con	Stat	2	20.9	46
227	F	Con	Stat	3	38.9	22
313	F	Con	Stat	21	30.4	26
365	F	Con	Stat	21	25	47
179	F	Con	VEx	83	24.7	76
163	F	Con	VEx	93	34.3	37
193	F	Con	VEx	82	31.3	32
214	F	Con	VEx	2	17.6	20
312	F	Con	VEx	21	30	23
318	F	Con	VEx	21	27.5	43
349	F	Con	VEx	24	25	27
350	F	Con	VEx	21	40.5	40
389	F	Con	VEx	54	32.5	43
113	F	AIE	Stat	91	22.2	38
139	F	AIE	Stat	79	26.2	68
168	F	AIE	Stat	93	29.2	51
175	F	AIE	Stat	83	12.8	89
187	F	AIE	Stat	82	45.8	59
199	F	AIE	Stat	1	46.2	29
222	F	AIE	Stat	3	37.1	38
230	F	AIE	Stat	4	40	28
322	F	AIE	Stat	24	59.5	45
364	F	AIE	Stat	21	36.4	36
387	F	AIE	Stat	54	25	39
186	F	AIE	VEx	82	20.5	42
101	F	AIE	VEx	6	10.3	32
134	F	AIE	VEx	81	20.3	82
140	F	AIE	VEx	79	25.6	46
167	F	AIE	VEx	93	28.2	49
198	F	AIE	VEx	1	29.4	20
379	F	AIE	VEx	53	34.4	35
380	F	AIE	VEx	54	30	43
114	M	Con	Stat	91	29.3	44
149	M	Con	Stat	69	13.8	61
178	M	Con	Stat	83	28.6	45
201	M	Con	Stat	1	40	18
216	M	Con	Stat	2	21.7	26
223	M	Con	Stat	3	17.8	31
233	M	Con	Stat	4	40.9	25
331	M	Con	Stat	23	45	23
361	M	Con	Stat	24	18.5	68

345	M	Con	Stat	24	50	23
311	M	Con	Stat	21	33.3	30
116	M	Con	VEx	91	45.5	14
217	M	Con	VEx	2	17.6	20
189	M	Con	VEx	82	31.4	38
306	M	Con	VEx	12	25	19
305	M	Con	VEx	12	15.4	15
148	M	Con	VEx	69	30.4	26
202	M	Con	VEx	1	14.3	38
154	M	AIE	Stat	69	38.5	26
159	M	AIE	Stat	93	21.6	40
172	M	AIE	Stat	83	30	33
229	M	AIE	Stat	3	30	13
131	M	AIE	Stat	81	41.4	32
212	M	AIE	Stat	2	10	13
307	M	AIE	Stat	12	26.1	26
362	M	AIE	Stat	24	34.1	44
118	M	AIE	VEx	91	22.2	30
132	M	AIE	VEx	81	21.2	36
145	M	AIE	VEx	79	62.5	11
160	M	AIE	VEx	93	36.4	14
184	M	AIE	VEx	82	15.4	29
197	M	AIE	VEx	1	10	11
308	M	AIE	VEx	12	20.6	37
309	M	AIE	VEx	12	11.4	38
383	M	AIE	VEx	53	36.4	36

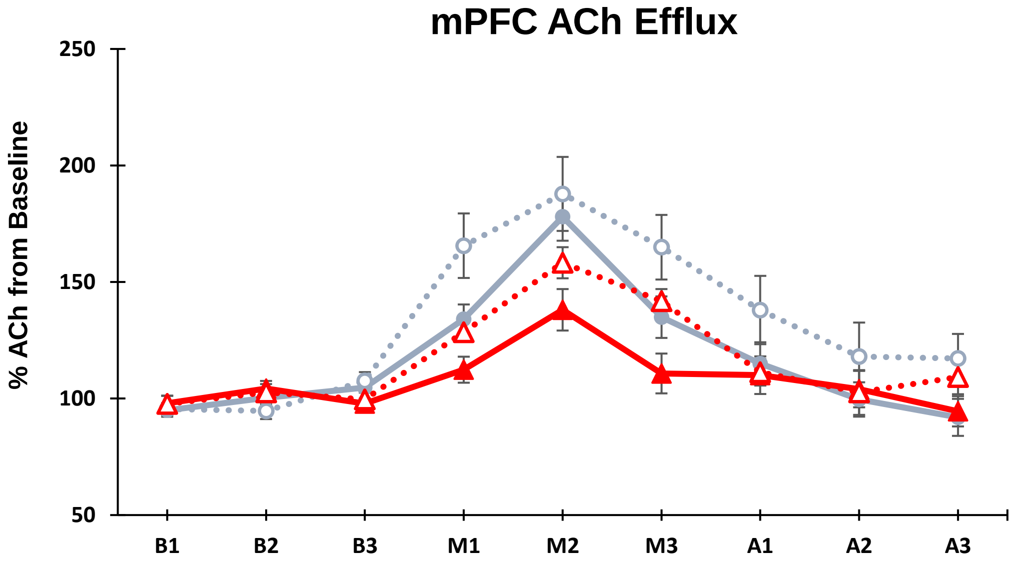

mPFC ACh Efflux
% ACh from Baseline
250
200
150
100
50
B1
B2
B3
M1
M2
M3
A1
A2
A3

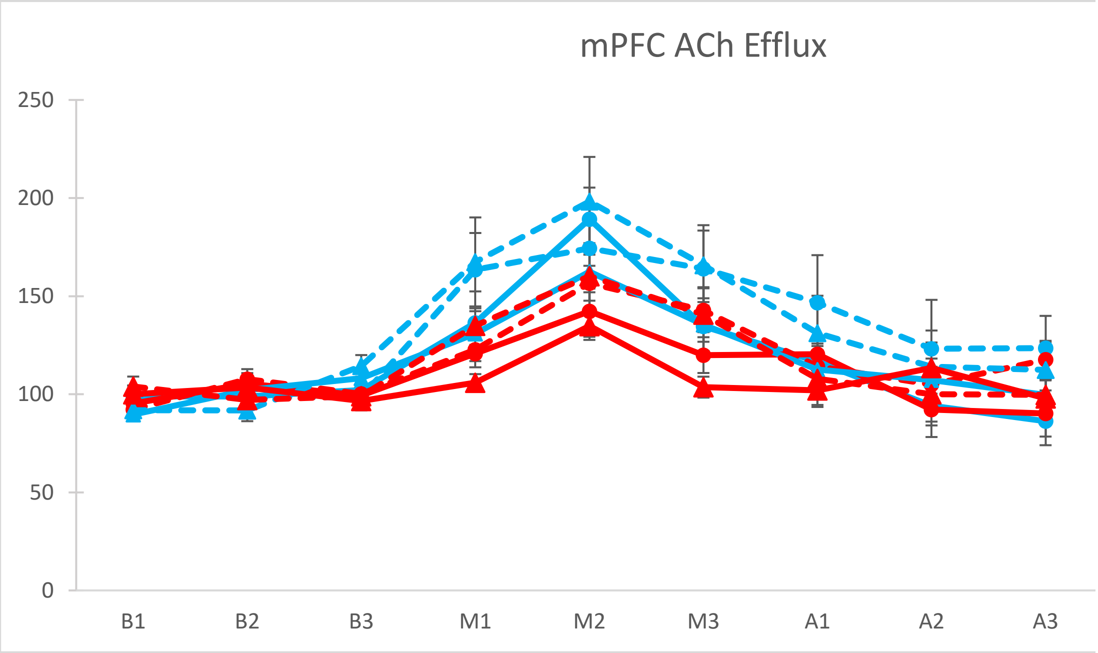

mPFC ACh Efflux
250
200
150
100
50
0
B1
B2
B3
M1
M2
M3
A1
A2
A3

Raw Data

Animal ID	Sex	Treatment	Exercise	B1	B2	B3	M1	M2
114	Male	Con	Stat	95.1183	97.7855	107.096	129.69	134.495
149	Male	Con	Stat	102.217	97.7826	100	104.869	205.172
178	Male	Con	Stat	84.2156	115.784	100	140.303	173.489
201	Male	Con	Stat	96.65	103.35	100	127.714	194.703
216	Male	Con	Stat	93.6058	106.11	100.284	110.227	110.354
223	Male	Con	Stat	113.828	104.948	81.2243	121.16	282.21
233	Male	Con	Stat	95.2068	104.793	100	120.522	199.502
331	Male	Con	Stat	114.5638	65.99865	119.4375	168.7681	167.2022
361	Male	Con	Stat	95.412404	100	104.5876	182.31252	280.58319
345	Male	Con	Stat	76.7044	110.0169	113.2787	136.2494	159.8837
311	Male	Con	Stat	120.8361	81.56355	97.60033	159.0998	174.0283
116	Male	Con	Vex	96.8257	103.174	100	88.6973	157.514
217	Male	Con	Vex	112.351	87.6486	100	106.16	134.77
189	Male	Con	Vex	95.0067	104.993	100	126.289	172.001
306	Male	Con	Vex	118.7447	83.98534	97.26994	204.1364	130.7521
305	Male	Con	Vex	89.554698	110.4453	100	275.51451	282.71586
148	Male	Con	Vex	100	96.223234	84.335899	119.44087	118.64474
202	Male	Con	Vex	88.96702	102.29551	108.73747	223.46361	224.16969
154	Male	AIE	Stat	71.7041	128.296	100	97.7287	151.812
159	Male	AIE	Stat	107.848	92.1517	100	104.869	178.049
172	Male	AIE	Stat	111.323	110.55	78.1268	149.023	182.586
229	Male	AIE	Stat	82.2234	115.525	102.251	119.463	113.051
131	Male	AIE	Stat	114.384	85.6157	100	111.287	116.778
307	Male	AIE	Stat	88.54829	102.6	108.8517	122.3862	94.22078
362	Male	AIE	Stat	90.866813	102.78165	106.35154	140.36184	159.43101
118	Male	AIE	Vex	92.156	107.844	100	130.268	197.14
132	Male	AIE	Vex	104.183	95.8173	100	119.577	143.884
145	Male	AIE	Vex	103.818	96.1825	100	107.625	147.634
160	Male	AIE	Vex	76.1507	123.849	100	112.135	158.594

184	Male	AIE	Vex	90.3173	109.683	100	90.3259	197.842
197	Male	AIE	Vex	79.8797	140.983	79.137	135.445	169.126
308	Male	AIE	Vex	98.6701	99.38098	101.9489	144.2731	127.2087
309	Male	AIE	Vex	94.60143	97.71967	107.6789	122.5146	138.6447
383	Male	AIE	Vex	90.1239	97.12745	112.7486	142.4113	128.9906
123	Female	Con	Stat	85.1174	114.883	100	125.352	151.697
128	Female	Con	Stat	92.0818	107.918	100	135.644	206.296
137	Female	Con	Stat	82.5889	101.441	115.97	130.29	149.539
144	Female	Con	Stat	88.2918	111.708	100	158.32	162.426
192	Female	Con	Stat	106.641	77.4508	115.908	121.857	132.719
213	Female	Con	Stat	84.8319	97.6933	117.475	155.779	182.95
227	Female	Con	Stat	99.1466	100.853	100	60.1633	174.794
365	Female	Con	Stat	78.340319	105.23283	116.42685	159.56965	139.3919
179	Female	Con	Vex	102.606	73.7225	123.671	142.285	182.764
163	Female	Con	Vex	77.901783	77.733746	144.36447	205.60122	305.0243
193	Female	Con	Vex	93.9687	106.031	100	125.091	246.7
214	Female	Con	Vex	83.1466	95.8873	120.966	258.867	303.401
312	Female	Con	Vex	86.40091	100.4444	113.1547	165.0224	141.7102
318	Female	Con	Vex	100	77.632208	104.59074	117.77705	146.9487
349	Female	Con	Vex	90.268937	123.81107	85.919996	154.6185	154.1318
350	Female	Con	Vex	94.066066	89.884408	116.04953	191.29735	150.45216
389	Female	Con	Vex	98.06523	80.69107	121.2437	145.1592	153.1316
113	Female	AIE	Stat	128.0554	71.9446	100	106.318	105.355
139	Female	AIE	Stat	89.4372	110.563	100	94.3744	124.435
175	Female	AIE	Stat	93.2508	110.953	95.7964	97.5319	142.52
187	Female	AIE	Stat	105.37	126.579	68.0513	89.1093	141.53
199	Female	AIE	Stat	101.743	116.36	81.8971	95.0545	174.568
222	Female	AIE	Stat	112.739	98.1886	89.0726	114.008	121.122
230	Female	AIE	Stat	86.399	103.312	110.289	129.048	141.637
364	Female	AIE	Stat	97.785533	94.686274	107.52819	124.14613	154.66716
322	Female	AIE	Stat	85.18184	98.21016	116.608	103.1362	107.3999
186	Female	AIE	Vex	85.6288	102.635	111.736	109.211	147.378
101	Female	AIE	Vex	94.14662	105.8534	100	126.9872	136.5057
134	Female	AIE	Vex	119.926	100.309	79.7644	167.839	179.139
140	Female	AIE	Vex	87.5543	109.441	103.005	171.201	178.304
167	Female	AIE	Vex	109.992	90.008	100	143.671	155.8
198	Female	AIE	Vex	102.84	90.2788	106.881	107.513	259.166
380	Female	AIE	Vex	105.8971	93.34792	100.755	101.2488	117.0928
379	Female	AIE	Vex	125.3247	84.96635	89.7089	152.0383	108.1416

Repeated measures ANOVA
Treatment, Exercise, and Sex as between-subjects factors
Phase as a within-subjects factor
Sphericity assumption violated ($p < 0.0001$)
Greenhouse Geisser Correction Applied
Significant Phase * Treatment effect ($p = 0.003$)
Significant Phase * Exercise effect ($p = 0.030$)
Figures presented with males and females either separate or together

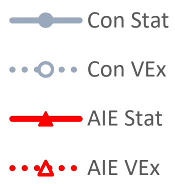

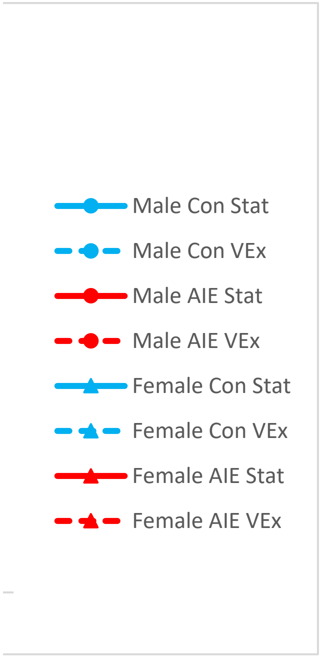

Measure:

Within Subjects Effect
Phase

Tests the null hypoth
a. Design: Intercept +
b. May be used to ad

Measure:

Source
Phase
Phase * Sex
Phase * Treatment
Phase * Exercise

M3	A1	A2	A3
128.474	108.242	89.9705	132.904
137.931	133.067	134.086	64.4145
84.8003	73.9576	43.0473	20.1527
107.56	124.472	141.503	108.789
106.94	71.9825	73.8463	72.5023
122.453	92.06	72.4659	34.6936
183.303	206.024	115.493	157.491
157.5201	143.8723	77.83363	90.58856
89.253759	81.939295	111.68405	113.7799
137.9333	93.90428	52.00219	72.95323
223.6102	154.1026	123.2047	80.44293
143.373	166.865	131.419	189.342
116.513	86.7602	72.8716	90.6651
157.668	147.313	152.585	167.836
146.6313	140.0025	128.6059	144.4614
274.14951	274.38672	244.8673	104.81097
138.34148	85.087561	91.462427	85.789728
171.1957	126.52913	40.116228	81.548743
118.335	133.816	44.3635	45.7947
129.288	112.958	146.737	98.7581
132.057	119.296	86.0736	102.482
105.481	108.574	58.8042	63.6579
85.0656	100.072	83.8691	74.8172
108.3035	129.1008	133.0024	139.2447
161.10383	139.19755	91.974557	106.53836
192.249	158.583	84.3766	152.051
136.351	126.269	101.869	105.562
134.925	115.068	80.3721	86.9748
102.467	67.028	92.6617	85.9907

Phase * Sex * Treatment
Phase * Sex * Exercise
Phase * Treatment * Exercise
Phase * Sex * Treatment * Exercise
Error(Phase)

Average

	Con	Stat
	Con	VEx
	AIE	Stat
	AIE	VEx

St Dev

	Con	Stat
	Con	VEx
	AIE	Stat
	AIE	VEx

SEM

96.4927	72.1532	74.0739	113.481		Con	Stat
148.864	152.037	155.606	166.761		Con	VEx
155.0987	113.3018	125.9335	103.5213		AIE	Stat
128.3693	117.0276	124.4172	141.6569		AIE	VEx
190.5363	106.9649	104.5375	102.1101			
119.617	75.6074	49.8179	54.907			
108.365	134.993	72.2116	70.3119	**Average**		
92.507	97.2208	99.0735	110.565			
133.795	75.8857	116.101	101.881	Male	Con	Stat
123.698	134.097	123.856	105.39	Male	Con	VEx
200.712	193.724	130.562	107.156	Male	AIE	Stat
118.617	73.35876	131.574	118.885	Male	AIE	VEx
186.45668	115.7446	134.06041	128.72105	Female	Con	Stat
				Female	Con	VEx
100.984	69.4697	69.5883	72.8509	Female	AIE	Stat
257.7892	153.6905	122.57012	144.02	Female	AIE	VEx
198.323	199.008	217.055	178.016			
250.938	241.282	168.991	130.969	**St Dev**		
151.2609	109.4435	52.71227	127.3219	Male	Con	Stat
112.61119	90.952384	58.86309	32.332186	Male	Con	VEx
118.04886	129.04725	146.50545	94.046688	Male	AIE	Stat
192.28267	85.432845	104.06923	102.28439	Male	AIE	VEx
108.8034	102.306	86.12751	129.354	Female	Con	Stat
				Female	Con	VEx
93.2118	89.317	101.064	69.076	Female	AIE	Stat
117.627	97.5065	81.7563	58.5182	Female	AIE	VEx
106.168	81.0034	42.0394	79.8398			
109.565	104.078	115.831	99.1261	**SEM**		
135.069	160.873	125.976	79.5735	Male	Con	Stat
87.2293	103.374	169.767	158.956	Male	Con	VEx
81.4054	64.2329	87.5472	110.257	Male	AIE	Stat
91.91671	103.03963	125.82273	101.16892	Male	AIE	VEx
110.2903	113.819	170.08	124.673	Female	Con	Stat
				Female	Con	VEx
98.0185	72.891	78.5221	99.1387	Female	AIE	Stat
138.9529	66.72108	35.05414	135.6262	Female	AIE	VEx
148.119	182.347	125.115	131.498			
202.205	92.1343	138.899	88.5866			
91.1927	98.0038	92.0549	77.7733			
188.323	139.669	154.948	96.0704			
119.4917	98.20903	107.0537	89.36437			
138.9336	112.92	68.51812	80.02199			

Mauchly's Test of Sphericity[a]

Mauchly's W	Approx. Chi-Square	df	Sig.	Epsilon[b] Greenhouse-Geisser	Huynh-Feldt	Lower-bound
0.075	148.174	35	0.000	0.635	0.782	0.125

esis that the error covariance matrix of the orthonormalized transformed dependent
- Sex + Treatment + Exercise + Sex * Treatment + Sex * Exercise + Treatment * Exercise
just the degrees of freedom for the averaged tests of significance. Corrected tests are

Tests of Within-Subjects Effects

	Type III Sum of Squares	df	Mean Square	F	Sig.	Partial Eta Squared
Sphericity Assumed	284329.481	8	35541.185	42.101	0.000	0.412
Greenhouse-Geisser	284329.481	5.082	55944.606	42.101	0.000	0.412
Huynh-Feldt	284329.481	6.257	45444.562	42.101	0.000	0.412
Lower-bound	284329.481	1.000	284329.481	42.101	0.000	0.412
Sphericity Assumed	3098.539	8	387.317	0.459	0.885	0.008
Greenhouse-Geisser	3098.539	5.082	609.668	0.459	0.810	0.008
Huynh-Feldt	3098.539	6.257	495.241	0.459	0.846	0.008
Lower-bound	3098.539	1.000	3098.539	0.459	0.501	0.008
Sphericity Assumed	24484.068	8	3060.509	3.625	0.000	0.057
Greenhouse-Geisser	24484.068	5.082	4817.480	3.625	0.003	0.057
Huynh-Feldt	24484.068	6.257	3913.304	3.625	0.001	0.057
Lower-bound	24484.068	1.000	24484.068	3.625	0.062	0.057
Sphericity Assumed	16889.638	8	2111.205	2.501	0.011	0.040
Greenhouse-Geisser	16889.638	5.082	3323.201	2.501	0.030	0.040

Huynh-Feldt	16889.638	6.257	2699.482	2.501	0.020	0.040
Lower-bound	16889.638	1.000	16889.638	2.501	0.119	0.040
Sphericity Assumed	2713.653	8	339.207	0.402	0.920	0.007
Greenhouse-Geisser	2713.653	5.082	533.938	0.402	0.850	0.007
Huynh-Feldt	2713.653	6.257	433.725	0.402	0.884	0.007
Lower-bound	2713.653	1.000	2713.653	0.402	0.529	0.007
Sphericity Assumed	11160.106	8	1395.013	1.652	0.108	0.027
Greenhouse-Geisser	11160.106	5.082	2195.860	1.652	0.145	0.027
Huynh-Feldt	11160.106	6.257	1783.727	1.652	0.128	0.027
Lower-bound	11160.106	1.000	11160.106	1.652	0.204	0.027
Sphericity Assumed	4305.698	8	538.212	0.638	0.746	0.011
Greenhouse-Geisser	4305.698	5.082	847.188	0.638	0.674	0.011
Huynh-Feldt	4305.698	6.257	688.183	0.638	0.707	0.011
Lower-bound	4305.698	1.000	4305.698	0.638	0.428	0.011
Sphericity Assumed	2831.946	8	353.993	0.419	0.910	0.007
Greenhouse-Geisser	2831.946	5.082	557.213	0.419	0.838	0.007
Huynh-Feldt	2831.946	6.257	452.632	0.419	0.873	0.007
Lower-bound	2831.946	1.000	2831.946	0.419	0.520	0.007
Sphericity Assumed	405214.402	480	844.197			
Greenhouse-Geisser	405214.402	304.940	1328.832			
Huynh-Feldt	405214.402	375.397	1079.428			
Lower-bound	405214.402	60.000	6753.573			

B1	B2	B3	M1	M2	M3	A1
95.02094334	100.2795857	104.6994	134.0994614	177.9703	134.923492	114.9608
95.49212776	94.66266778	107.519	165.5887761	187.8019	164.932075	137.9735
97.92871103	104.2697926	97.80148	112.3653229	138.0726	110.757277	110.0161
97.71821471	102.6721394	99.60963	128.4873059	158.2701	141.799376	111.2546
12.36392935	12.72587653	9.567387	27.33136389	44.67977	38.8568666	40.00227
10.25909819	13.9022718	15.26204	55.38179544	63.49321	55.4474513	58.62894
14.62220963	14.56646458	12.7769	17.27373485	27.52372	21.3261161	23.42317
13.17589835	13.51671029	9.246732	23.02517108	36.69623	35.2218377	33.27408

2.836479924	2.919516304	2.194909	6.270244905	10.25024	8.91437658	9.177151
2.564774547	3.475567951	3.81551	13.84544886	15.8733	13.8618628	14.65724
3.546406753	3.532886591	3.098854	4.189496077	6.675482	5.17234289	5.680952
3.195624742	3.278283779	2.242662	5.584424258	8.900144	8.54255043	8.070149

B1	B2	B3	M1	M2	M3	A1
98.94165491	98.92110909	102.1371	136.4468018	189.2384	134.525333	116.6931
100.2071169	98.394998	98.62047	163.3859561	174.3668	163.981713	146.7063
95.2710862	105.3600069	99.36872	120.7312481	142.2754	119.947704	120.4306
92.21112556	107.6207667	100.1682	122.7305444	156.5627	142.817	114.2703
89.62996493	102.147491	108.2225	130.8718683	162.4766	135.47096	112.5789
91.82491398	91.75974428	114.44	167.3020806	198.2515	165.671246	131.1814
99.99575256	103.4218482	96.58251	105.8584922	134.8038	103.609168	101.9159
103.91369	97.10493375	98.98129	134.9636625	160.1909	140.65455	107.8619
13.19790636	13.92298243	9.662634	24.4340651	53.31317	41.3036081	40.83814
11.32439031	9.614539193	7.255331	70.71456309	59.27978	51.4219552	63.96808
16.18324613	14.34622768	9.995073	18.52949235	34.40781	24.1244608	14.25201
9.639921344	15.51969746	9.085021	17.57263479	26.68301	33.5316133	30.82641
9.320953344	11.51617372	8.802993	32.36373152	24.47454	38.0101256	41.48553
8.159592266	16.47386768	16.55566	44.64228205	68.13589	61.4814538	57.07426
13.90977962	15.54449986	15.07797	13.89561831	22.47256	16.8782551	26.6017
14.41338911	8.684837394	10.01261	27.71886367	47.50068	39.3418859	37.68015
3.979318493	4.197937152	2.913394	7.367147823	16.07453	12.4535064	12.31316
4.280217217	3.633954239	2.742258	26.72759257	22.40565	19.4356722	24.17766
6.116692097	5.422364384	3.777782	7.003489812	13.00493	9.1181891	5.386752
3.213307115	5.173232486	3.02834	5.857544929	8.894337	11.1772044	10.27547
3.295454658	4.071582267	3.112328	11.44230701	8.653055	13.4386088	14.66735
2.719864089	5.491289226	5.518553	14.88076068	22.71196	20.4938179	19.02475
4.398658534	4.915602463	4.768074	4.394180336	7.106448	5.33737289	8.412196
5.095902588	3.070553707	3.539991	9.800098234	16.79403	13.9094572	13.32194

A2	A3
99.59963	91.92256
118.0256	117.2281
104.0442	94.53009
102.589	109.1876
32.13557	34.61907
58.28057	41.96224
39.61664	30.21879
32.18375	26.7817

7.372406	7.942159
14.57014	10.49056
9.608448	7.329133
7.805705	6.495516

A2	A3
94.10332	86.24652
123.1325	123.4934
92.11777	90.18471
104.8719	117.5676
107.1571	99.72712
114.0536	112.355
113.3204	97.90984
100.0206	99.75995

33.16378	40.54606
66.08869	43.49255
36.85483	31.11633
26.28353	28.98163
31.16987	24.7592
55.24777	42.68245
41.26499	30.92995
39.5446	22.08297

9.999257	12.2251
24.97918	16.43864
13.92982	11.76087
8.761176	9.660542
11.02021	8.753699
18.41592	14.22748
13.04914	9.780908
13.98113	7.807508

Animal ID	Sex	Treatment	Gavage1	Gavage2	Gavage3	Gavage4	Gavage5	Gavage6	Gavage7
201	Male	Con	90	95	123	128	147	149	192
202	Male	Con	83	92	115	122	145	152	191
203	Male	Con	69	76	96	101	124	133	168
204	Male	Con	96	103	129	133	158	169	203
217	Male	Con	88	93	116	121	146	156	180
218	Male	Con	101	106	113	141	164	175	194
219	Male	Con	68	75	94	101	124	131	158
220	Male	Con	77	84	107	114	136	143	171
233	Male	Con	71	78	94	105	128	135	160
234	Male	Con	87	94	119	127	149	160	183
235	Male	Con	68	72	94	99	120	127	150
236	Male	Con	88	94	118	128	148	157	185
253	Male	Con	85	91	114	122	145	156	181
254	Male	Con	69	74	96	99	120	128	152
255	Male	Con	96	102	124	131	155	165	187
262	Male	Con	62	68	84	85	104	112	128
101	Male	Con	88	93	111	121	144	150	178
102	Male	Con	73	78	99	106	126	140	167
117	Male	Con	68	73	93	98	114	125	147
118	Male	Con	74	79	96	106	130	138	158
125	Male	Con	77	83	104	110	138	148	164
126	Male	Con	68	74	94	99	120	130	160
129	Male	Con	79	86	106	113	143	148	174
130	Male	Con	72	80	98	106	132	138	163
137	Male	Con	71	76	89	102	122	128	148
138	Male	Con	69	77	96	103	128	131	158
213	Fema	Con	75	78	97	103	121	126	143
214	Fema	Con	93	99	121	126	140	144	163
215	Fema	Con	91	98	120	124	134	142	148
216	Fema	Con	87	88	106	110	127	133	148
229	Fema	Con	85	91	110	117	128	135	149
230	Fema	Con	59	64	80	87	99	108	122
231	Fema	Con	62	66	82	86	101	109	125
232	Fema	Con	69	75	92	95	111	118	135
245	Fema	Con	67	74	89	95	108	113	131
246	Fema	Con	87	91	112	115	131	136	150
247	Fema	Con	63	66	82	86	103	108	127
248	Fema	Con	59	67	82	87	103	108	126
249	Fema	Con	83	89	105	111	127	134	149
250	Fema	Con	86	93	109	113	130	135	148
258	Fema	Con	64	70	85	90	108	113	132
259	Fema	Con	91	98	118	123	135	140	152
105	Fema	Con	55	61	72	76	94	99	117
106	Fema	Con	83	89	104	113	132	138	153
109	Fema	Con	62	65	81	85	103	111	127
110	Fema	Con	83	87	104	110	128	137	152

113	Fema	Con	62	68	85	92	105	117	135
114	Fema	Con	63	68	81	86	107	111	128
121	Fema	Con	69	72	88	92	112	116	134
122	Fema	Con	64	68	83	89	106	108	126
133	Fema	Con	60	65	77	84	103	105	118
134	Fema	Con	60	66	80	82	102	110	125
139	Fema	Con	72	74	93	98	116	119	141
209	Male	AIE	63	64	81	84	98	106	130
210	Male	AIE	69	73	94	91	116	122	148
211	Male	AIE	84	87	110	113	137	143	168
212	Male	AIE	77	82	105	110	134	141	166
225	Male	AIE	78	84	105	110	135	140	164
226	Male	AIE	67	71	93	98	120	129	150
227	Male	AIE	78	80	101	106	128	136	157
228	Male	AIE	89	90	118	121	141	151	169
241	Male	AIE	97	103	126	134	159	161	188
242	Male	AIE	68	71	93	99	120	128	151
243	Male	AIE	93	95	115	121	140	143	163
260	Male	AIE	65	68	88	94	112	117	132
261	Male	AIE	61	64	79	82	98	103	120
263	Male	AIE	61	67	80	83	99	107	122
103	Male	AIE	71	74	91	96	111	120	146
104	Male	AIE	93	97	115	126	149	158	184
119	Male	AIE	74	78	100	105	128	135	158
120	Male	AIE	72	75	93	97	118	126	145
127	Male	AIE	68	74	92	95	118	124	149
128	Male	AIE	80	81	102	109	131	142	166
131	Male	AIE	60	64	79	86	108	114	135
132	Male	AIE	74	80	99	105	129	136	160
141	Male	AIE	68	73	93	102	125	134	163
142	Male	AIE	70	75	94	102	124	132	155
205	Fema	AIE	60	66	79	83	98	105	121
206	Fema	AIE	64	69	82	90	106	113	139
207	Fema	AIE	84	87	106	112	123	130	139
208	Fema	AIE	81	87	101	110	125	131	143
221	Fema	AIE	69	72	91	93	113	119	136
222	Fema	AIE	69	71	87	91	109	114	125
223	Fema	AIE	63	65	80	84	102	109	127
224	Fema	AIE	59	64	79	85	102	105	124
237	Fema	AIE	68	71	90	93	110	116	132
238	Fema	AIE	61	64	74	77	93	96	115
239	Fema	AIE	85	88	104	107	124	124	140
240	Fema	AIE	66	68	84	86	102	112	123
251	Fema	AIE	64	69	86	89	110	114	136
252	Fema	AIE	72	75	95	99	119	124	145
256	Fema	AIE	68	61	82	89	110	114	136
257	Fema	AIE	96	83	102	109	131	136	157

107	Fema	AIE	82	85	98	103	121	127	145
108	Fema	AIE	65	68	82	87	100	105	122
111	Fema	AIE	83	81	99	103	121	127	144
112	Fema	AIE	65	68	85	88	99	106	122
115	Fema	AIE	62	62	78	83	102	105	124
116	Fema	AIE	63	70	82	84	100	108	123
123	Fema	AIE	67	69	81	88	100	110	125
124	Fema	AIE	68	69	84	90	107	108	130
135	Fema	AIE	69	73	86	92	112	117	135
136	Fema	AIE	56	58	72	81	98	102	118
140	Fema	AIE	72	75	87	94	113	117	137

Gavage8	Gavage9	Gavage10	Gavage11	Gavage12	Gavage13	Gavage14	Gavage15	Gavage16
194	203	206	221	230	244	258	272	268
182	206	211	235	238	261	271	293	293
160	183	182	217	220	245	261	282	283
203	222	222	249	253	274	288	304	304
183	206	212	225	236	254	264	283	290
199	222	228	243	251	278	283	295	304
165	190	193	212	217	234	245	268	268
178	202	209	230	239	255	260	291	295
165	187	193	206	212	232	244	264	264
190	215	221	245	250	271	283	302	313
159	184	186	206	216	238	243	264	267
189	220	226	240	247	268	281	303	306
188	212	221	242	248	275	283	310	313
158	184	191	217	221	239	252	271	277
192	218	222	240	246	276	280	304	314
133	147	146	158	159	174	176	185	191
183	208	212	233	242	268	271	209	292
176	197	204	230	230	252	259	281	283
150	172	179	203	205	222	236	248	256
164	191	197	216	222	241	250	265	274
175	199	202	225	227	255	258	287	290
166	185	193	217	222	248	251	276	276
180	206	209	234	243	267	272	291	297
170	203	203	230	235	259	260	290	289
155	180	185	208	220	235	240	255	270
166	187	192	222	226	251	255	273	283
147	159	159	167	174	185	187	199	202
163	169	173	185	186	195	194	212	205
156	163	164	172	169	178	181	186	188
145	162	170	178	183	194	201	206	210
147	159	163	166	166	177	177	190	192
126	140	144	158	158	177	181	174	177
128	144	150	154	157	174	168	187	190
139	154	154	162	167	177	175	189	188
137	146	153	164	166	174	177	189	187
155	166	170	172	166	180	177	199	201
129	146	148	155	160	164	172	180	183
130	151	155	165	165	168	171	183	187
150	159	164	173	178	179	184	186	193
151	163	164	180	181	190	194	204	205
140	154	162	175	175	180	184	192	195
155	167	170	179	177	185	191	199	205
122	134	136	149	152	161	164	176	170
155	174	174	192	195	214	219	231	237
133	145	146	165	166	181	186	193	191
149	170	169	185	188	205	202	216	218

138	151	156	165	171	180	183	200	198
128	147	142	158	158	175	176	190	198
138	158	160	173	173	185	187	203	207
127	144	149	162	169	181	177	187	191
125	142	142	151	156	164	170	179	186
127	143	143	160	165	183	183	185	189
141	164	162	173	169	187	187	207	212
133	156	159	182	185	198	207	234	237
154	181	183	202	207	222	232	252	253
170	186	197	207	214	238	243	267	275
171	193	197	216	217	237	241	266	271
167	184	193	212	209	236	243	264	265
155	179	181	194	220	218	232	246	258
163	172	175	196	202	217	226	241	248
178	199	196	210	223	236	245	265	272
192	214	215	229	248	263	265	280	291
158	183	184	209	218	233	241	260	267
168	187	192	207	214	230	248	257	262
139	153	156	165	170	184	188	203	199
123	135	141	147	150	161	165	174	177
126	147	149	166	167	172	176	189	190
154	174	173	196	203	222	231	252	249
183	216	219	246	252	275	278	300	302
155	184	184	209	214	239	239	259	268
152	174	172	194	200	218	226	247	245
157	179	188	176	182	191	196	220	222
161	187	192	212	211	237	242	264	265
139	161	164	150	193	214	214	237	241
164	186	192	160	217	244	241	261	264
170	192	194	218	222	247	250	274	276
161	179	183	204	207	230	233	251	253
125	138	142	158	158	159	164	167	171
135	155	155	168	177	178	184	187	194
146	154	155	160	168	174	183	187	185
148	161	168	175	180	182	193	205	198
137	152	154	166	167	175	185	190	197
133	141	147	150	152	164	172	172	181
129	144	147	160	167	174	184	189	198
124	142	142	149	163	160	169	174	181
135	151	153	163	175	180	186	194	206
121	139	140	154	166	162	171	170	178
144	156	159	164	171	175	175	182	188
128	143	148	240	167	167	173	169	178
139	165	167	186	190	208	211	226	236
149	166	172	188	190	207	218	225	233
140	165	167	183	189	211	217	237	238
161	190	194	210	217	236	242	266	267

146	163	165	177	183	191	191	203	211
129	141	142	158	154	165	166	179	175
144	156	160	174	177	185	187	196	203
129	139	137	149	149	160	163	171	172
130	141	144	154	159	176	176	182	185
129	139	146	154	154	162	166	176	175
131	144	146	158	159	171	175	182	189
132	154	150	161	166	179	181	191	199
141	160	155	209	172	189	186	202	202
125	142	138	221	149	165	170	180	180
142	162	163	178	179	188	193	206	209

Animal ID	Sex	Treatment	Condition	Dam	BEC
101	Male	Con	Stat	1	N/A
102	Male	Con	Stat	3	N/A
103	Male	AIE	VEx	3	192
104	Male	AIE	VEx	1	180.3
105	Female	Con	VEx	3	N/A
106	Female	Con	VEx	1	N/A
109	Female	Con	Stat	3	N/A
110	Female	Con	Stat	1	N/A
111	Female	AIE	VEx	1	253.6
112	Female	AIE	VEx	3	179.2
113	Female	Con	VEx	3	N/A
114	Female	Con	VEx	4	N/A
115	Female	AIE	Stat	4	187.7
116	Female	AIE	Stat	3	114.4
117	Male	Con	Stat	3	N/A
118	Male	Con	Stat	2	N/A
123	Female	AIE	Stat	4	160.3
124	Female	AIE	Stat	2	193.4
125	Male	Con	VEx	2	N/A
126	Male	Con	VEx	3	N/A
127	Male	AIE	Stat	3	138.9
128	Male	AIE	Stat	2	274.5
129	Male	Con	VEx	2	N/A
130	Male	Con	VEx	4	N/A
131	Male	AIE	Stat	4	172
132	Male	AIE	Stat	2	180.3
133	Female	Con	Stat	4	N/A
134	Female	Con	Stat	2	N/A
135	Female	AIE	VEx	2	205.4
136	Female	AIE	VEx	4	210.3
141	Male	AIE	VEx	4	156.1
142	Male	AIE	VEx	4	159.1
201	Male	Con	Stat	31	N/A
202	Male	Con	Stat	36	N/A
203	Male	Con	VEx	35	N/A
204	Male	Con	VEx	36	N/A
205	Female	AIE	Stat	32	251.6
206	Female	AIE	Stat	35	275.6
207	Female	AIE	VEx	31	238.6
208	Female	AIE	VEx	34	193.8
209	Male	AIE	VEx	32	176.6
210	Male	AIE	VEx	35	261.4
211	Male	AIE	Stat	34	276.3
212	Male	AIE	Stat	33	251.3
213	Female	Con	VEx	34	N/A
214	Female	Con	VEx	31	N/A

215	Female	Con	Stat	31	N/A
216	Female	Con	Stat	34	N/A
217	Male	Con	Stat	34	N/A
218	Male	Con	Stat	31	N/A
219	Male	Con	VEx	32	N/A
220	Male	Con	VEx	33	N/A
221	Female	AIE	Stat	33	172.7
222	Female	AIE	Stat	34	186.7
223	Female	AIE	VEx	35	161.7
224	Female	AIE	VEx	32	148.7
225	Male	AIE	Stat	33	245.1
226	Male	AIE	Stat	32	151.6
227	Male	AIE	VEx	33	220.5
228	Male	AIE	VEx	31	223.4
229	Female	Con	VEx	31	N/A
230	Female	Con	VEx	32	N/A
231	Female	Con	Stat	35	N/A
232	Female	Con	Stat	36	N/A
233	Male	Con	Stat	33	N/A
234	Male	Con	Stat	34	N/A
235	Male	Con	VEx	32	N/A
236	Male	Con	VEx	36	N/A
237	Female	AIE	Stat	36	194.8
238	Female	AIE	Stat	35	188.6
239	Female	AIE	VEx	36	141.1
240	Female	AIE	VEx	33	132.8
241	Male	AIE	VEx	31	136.4
242	Male	AIE	VEx	32	153.6
243	Male	AIE	Stat	31	231.5
245	Female	Con	VEx	33	N/A
246	Female	Con	VEx	36	N/A
247	Female	Con	Stat	32	N/A
248	Female	Con	Stat	35	N/A

Animal ID	Sex	Treatment	Condition	Dam	Cage running distance
101	Male	Con	Stat	1	N/A
102	Male	Con	Stat	3	N/A
103	Male	AIE	VEx	3	235061.2
104	Male	AIE	VEx	1	235061.2
105	Female	Con	VEx	3	568993.7
106	Female	Con	VEx	1	568993.7
109	Female	Con	Stat	3	N/A
110	Female	Con	Stat	1	N/A
111	Female	AIE	VEx	1	414524.0
112	Female	AIE	VEx	3	414524.0
113	Female	Con	VEx	3	408818.3
114	Female	Con	VEx	4	408818.3
115	Female	AIE	Stat	4	N/A
116	Female	AIE	Stat	3	N/A
117	Male	Con	Stat	3	N/A
118	Male	Con	Stat	2	N/A
123	Female	AIE	Stat	4	N/A
124	Female	AIE	Stat	2	N/A
125	Male	Con	VEx	2	289930.3
126	Male	Con	VEx	3	289930.3
127	Male	AIE	Stat	3	N/A
128	Male	AIE	Stat	2	N/A
129	Male	Con	VEx	2	262075.0
130	Male	Con	VEx	4	262075.0
131	Male	AIE	Stat	4	N/A
132	Male	AIE	Stat	2	N/A
133	Female	Con	Stat	4	N/A
134	Female	Con	Stat	2	N/A
135	Female	AIE	VEx	2	573933.8
136	Female	AIE	VEx	4	573933.8
141	Male	AIE	VEx	4	446788.1
142	Male	AIE	VEx	4	446788.1
201	Male	Con	Stat	31	N/A
202	Male	Con	Stat	36	N/A
203	Male	Con	VEx	35	270144.6
204	Male	Con	VEx	36	270144.6
205	Female	AIE	Stat	32	N/A
206	Female	AIE	Stat	35	N/A
207	Female	AIE	VEx	31	552893
208	Female	AIE	VEx	34	552893
209	Male	AIE	VEx	32	290849.9
210	Male	AIE	VEx	35	290849.9
211	Male	AIE	Stat	34	N/A
212	Male	AIE	Stat	33	N/A
213	Female	Con	VEx	34	585235.2
214	Female	Con	VEx	31	585235.2

215	Female	Con	Stat	31	N/A
216	Female	Con	Stat	34	N/A
217	Male	Con	Stat	34	N/A
218	Male	Con	Stat	31	N/A
219	Male	Con	VEx	32	304268.8
220	Male	Con	VEx	33	304268.8
221	Female	AIE	Stat	33	N/A
222	Female	AIE	Stat	34	N/A
223	Female	AIE	VEx	35	603934.1
224	Female	AIE	VEx	32	603934.1
225	Male	AIE	Stat	33	N/A
226	Male	AIE	Stat	32	N/A
227	Male	AIE	VEx	33	377295.6
228	Male	AIE	VEx	31	377295.6
229	Female	Con	VEx	31	801033.2
230	Female	Con	VEx	32	801033.2
231	Female	Con	Stat	35	N/A
232	Female	Con	Stat	36	N/A
233	Male	Con	Stat	33	N/A
234	Male	Con	Stat	34	N/A
235	Male	Con	VEx	32	301356
236	Male	Con	VEx	36	301356
237	Female	AIE	Stat	36	N/A
238	Female	AIE	Stat	35	N/A
239	Female	AIE	VEx	36	641224.1
240	Female	AIE	VEx	33	641224.1
241	Male	AIE	VEx	31	484413.6
242	Male	AIE	VEx	32	484413.6
243	Male	AIE	Stat	31	N/A
245	Female	Con	VEx	33	507125.3
246	Female	Con	VEx	36	507125.3
247	Female	Con	Stat	32	N/A
248	Female	Con	Stat	35	N/A
