# Supplementary material for: Voluntary wheel running exercise rescues behaviorally-evoked acetylcholine efflux in the medial prefrontal cortex and epigenetic changes in ChAT genes following adolescent intermittent ethanol exposure
Source: PLoS One. 2024 Oct 22;19(10):e0311405. doi: 10.1371/journal.pone.0311405 (PMC11495633; doi:10.1371/journal.pone.0311405)
Supplement: Attachment — Submitted filename: PONE-D-24-25335 - review 22.07.24 (1).docx [file pone.0311405.s004.docx]

PONE-D-24-25335

Voluntary wheel running exercise rescues behaviorally-evoked acetylcholine efflux in the medial prefrontal cortex and epigenetic changes in ChAT genes following adolescent intermittent ethanol exposure

This manuscript addresses a relevant issue regarding long-term deleterious effect of high ethanol exposure during adolescence upon cholinergic signaling and cognitive functions. Additionally, they employed voluntary activity later in life and investigated whether it might cause beneficial outcomes on ethanol-induced effects in their model. The study is strengthened by the inclusion of animals of both sexes as well as developmental measurement during the ethanol exposure period and at the time-point of behavioral evaluations. Although the experiments are pretty straight forward, there are some methodology, analysis and interpretive concerns. In the ensuing paragraphs these major and secondary concerns should be addressed and this information should be taken into consideration in the decision about this manuscript for publication.

Major concerns:

1 - To this reviewer, a matter of concern is the number of animals used in each experimental condition. The authors stated that, for both experiments, “no more than two pups of each sex per litter was assigned to an experimental condition”. This brings a concern about litter-effect on this manuscript’s data. Firstly, how many litters in each experiment were used?

It is an important issue since the variability, as well-known, within litters is lower than between. Thus, it is a major source of variability in an experiment and controlling it is crucial to enhance rigor and reproducibility. Thus, to avoid litter-effect on results the authors should use the mean of pups from each litter for each experimental condition. Accordingly, the sample size described in the manuscript should be corrected to present the adjusted numbers.

This brings another concern about whether the number of animals used was appropriated. In this sense, it is possible that some sample sizes be around 6, if using the mean per litter, which would be too small, particularly regarding behavioral evaluation.

To this reviewer it is critical that these questions are answered and that the answers reflect a reasonable experimental design.

2 – I understood that in experiment 1 were submitted to both behavioral tests. If that is correct, it is not clear why the sample size for the attention set shifting and reversal learning is bigger than in microdialysis and spontaneous alternation. Please, clarify it to the reader.

3 – It is not clear how animals used in the HPLC and cresyl violet staining were euthanized. Was it immediately after the attention set shifting and reversal learning test? What was the method used? Also, in line 243, it is described that, in the experiment 1, all rats were anaesthetized and perfused. Is that correct? Please, provide a detailed description for euthanasia in experiment 1.

4 – In some points of the manuscript the authors give the idea that previous data already shown that AIE model does not affect spontaneous alternation (lines 451-454). So, why did the authors used this test? Shouldn’t this be addressed as a limitation of the study?

5 – It is not clear to this reviewer why the authors presented the ChIP results separated by sex. In the statistical analysis, in the methods session, the authors stated that they used a 2 x 2 ANOVA. Thus, I assumed that sex was not a factor in this analysis. If that is corrected, the authors must provide rationale for, *a priori*, analyze males and females separately only for this variable. Otherwise, they should perform the analysis including sex as a factor. Please clarify it.

Minor:

1 – The description of the attention set shifting and reversal learning test could be improved if reorganized. For instance, in the second paragraph describes the acquisition session, which involves a response based on animal’s preferred side. The establishment of the side preference was not described yet. As a suggestion, considering the complexity of the protocol, the authors could describe it chronologically.

2 – The lines 269-272 should be revised.

3 – Some results are not addressed in the discussion session. For instance, was the sex difference in weight gain expected or not? What could explain it? Additionally, there was an effect of Exercise in the levels of H3K9me2 at Chat promoter of females (but, please, see comment 4 in major about the analysis of this variable). However, it is not discussed. Could it be related to the hyperactivity in females compared to males?

4 – The conclusion session should be revised. The authors wrote that: “… AIE, its effects on behaviors modulated by the cholinergic system are variable.”. It is difficult to conclude this since there are limitations in the behavioral testes used in this work (lines 451-454; 474-475; 488-495). Besides that, afterwards, the authors address phasic and tonic ACh signaling and attention set shifting behaviors. It was not addressed anywhere else in the manuscript. It is difficult to understand the impact/importance of these types of signaling to the study. If it is important, please provide rationale for it in the introduction and discussion sessions.
